# Exploring the genetic basis of fatty liver development in geese

**DOI:** 10.1038/s41598-020-71210-8

**Published:** 2020-08-31

**Authors:** Yunzhou Yang, Huiying Wang, Guangquan Li, Yi Liu, Cui Wang, Daqian He

**Affiliations:** 1 Institute of Animal Husbandry and Veterinary Science, Shanghai Academy of Agricultural Sciences, Shanghai, 201106 People’s Republic of China; 2 Department of Medical Biochemistry and Microbiology, Uppsala University, 75123 Uppsala, Sweden

**Keywords:** Genetics, Genomics, Comparative genomics

## Abstract

Although geese possess an adaptive physiological capacity for lipid storage, few candidate genes contributing to this ability are characterised. By comparing the genomes of individuals with extremely high and low fatty liver weights (FLW), candidate genes were identified, including *ARAP2*, *GABRE*, and *IL6*. Single-nucleotide polymorphisms in or near these genes were significantly (*p* < 0.05) associated with carcass traits (FLW) and biochemical indexes (very-low-density lipoprotein and N-terminal procollagen III), suggesting contribution to trait variation. A common variant at the 5′-end of *LCORL* explained ~ 18% and ~ 26% of the phenotypic variance in body weight with/without overfeeding and had significant effects on FLW (*p* < 0.01). *ZFF36L1*, *ARHGEF1* and *IQCJ,* involved in bile acid metabolism, blood pressure, and lipid concentration modulation, were also identified. The presence of highly divergent haplotypes within these genes suggested involvement in protection against negative effects from excessive lipids in the liver or circulatory system. Based on this and transcriptomic data, we concluded that geese hepatosteatosis results from severe imbalance between lipid accumulation and secretion, comparable to human non-alcohol fatty liver disease but involving other genes. Our results provided valuable insights into the genesis of geese fatty liver and detected potential target genes for treatment of lipid-related diseases.

## Introduction

Migratory birds are physiologically adapted to accumulating large amounts of lipids (more than 50% of their body weight) and efficiently utilising the stored fatty acids to endure flights of many days to arrive at their target habitats^1^. The domestic goose was domesticated from the greylag goose (*Anser Anser*) or swan goose (*Anser Cygnoides*), both of which are migratory birds, and its retained ability to accumulate lipids has been the foundation for the establishment of the fatty liver (*foie gras*) industry^2,3^. In selected geese, hepatic weight can increase more than eightfold in 2 weeks and account for more than 9% of the body weight^4^. Geese are considered an interesting model for understanding the pathogenesis of non-alcohol fatty liver disease (NAFLD) in human owing to multiple shared physiological properties^5^. NAFLD is an expanding health threat, with up to 25% global prevalence, and is becoming a heavy economic burden in many countries^6^. In the last decade, with the help of next-generation sequencing platforms, large-scale genome-wide association studies (GWAS) have led to the identification of an enormous number of sequence variations related to this chronic liver disease^7–10^. To date, more than five genes have been robustly demonstrated to be associated with NAFLD. These include *PNPLA3*, *TM6SF2*, *GCKR*, *MBOAT7*, and *HSD17B13*^11^. *PNPLA3* encodes the patatin-like phospholipase domain-containing 3 protein and has the most robust association with NAFLD: a common missense variant (rs738409, I148M) with NAFLD. This loss-of-function mutation disrupts the *PNPLA3* enzyme activity, resulting in impaired mobilisation of triglycerides from lipid droplets (LDs) and increased risk of steatohepatitis^9^. The protein encoded by *HSD17B13* gene is also located on LD surfaces, but mutations in this gene have no association with hepatic lipid content. The underlying mechanisms remain unexplained^7,12^. The exact function of the transmembrane 6 superfamily member 2 (*TM6SF2*) is not well understood, but it is involved in the enrichment of triglycerides to apolipoprotein B100 in very-low-density lipoprotein secretion from the hepatocyte. *GCKR* (glucokinase regulator) regulates de novo lipogenesis by controlling the influx of glucose in hepatocytes and a common missense loss-of-function (rs1260326) seems to represent the causal variant underlying the association with hepatic fat accumulation. The rs641738 C > T mutation close to the membrane-bound O-acyl-transferase domain-containing 7 (*MBOAT7*) is associated with a predisposition to accumulate fat in the liver and to develop NAFLD and inflammation, due to decreased protein expression^13^. Several other genes implicated in the regulation of lipid accumulation, inflammation, and fibrosis have also been identified as risk factors for NAFLD^14–16^.

Earlier studies on transcriptional or posttranscriptional regulation in overfed geese have attempted to reveal the biological mechanisms leading to fatty liver formation^3,17,18^. By comparing the transcriptome profiles of geese with and without overfeeding, significant increases in expression levels of genes involved in lipid synthesis (such as *DGAT2*, *FASN,* or *ELOVL6*) were detected; the altered expression levels resulted in excessive short-term lipid synthesis^3^. Meanwhile, suppression of genes functioning in lipid transportation and secretion, including *APOB,* was also observed. A recent study on miRNAs targeting *LPL* and *ELOVL6* in overfed geese indicated posttranscriptional regulation of genes associated with lipid metabolism^3,17^. In addition to genes related to lipid metabolism, downregulated genes involved in complementary systems were detected at 19 days in overfed geese. The downregulations are likely to suppress inflammation in hepatocytes when geese are subjected to severe hepatic steatosis^18^. Although these findings, at the transcriptional level, increased our understanding for the underpinnings of lipid metabolism in overfed geese, the specific genes responsible for fatty liver formation or lipid metabolism remain ambiguous.

In this study, we used genetics, genomics, and transcriptomics to identify genetic variants likely to contribute to high and low fatty liver weights in French Landes geese, which are famous for high susceptibility to steatosis. Several candidate genes involved in lipid metabolism (*GABRE* and *ESRRG*), inflammation and fibrosis (*IL6*), blood lipid regulation (*ZFP36L1* and *IQCJ*), and blood pressure (*ARHGEF1*) were identified. Genetic variants in candidate genes, for which birds with different liver weights displayed differences in allele frequencies, were tested for associations to blood lipid levels and fatty liver weights. Expression levels of candidate genes were explored.

## Results

### Phenotyping

**Figure 1:**
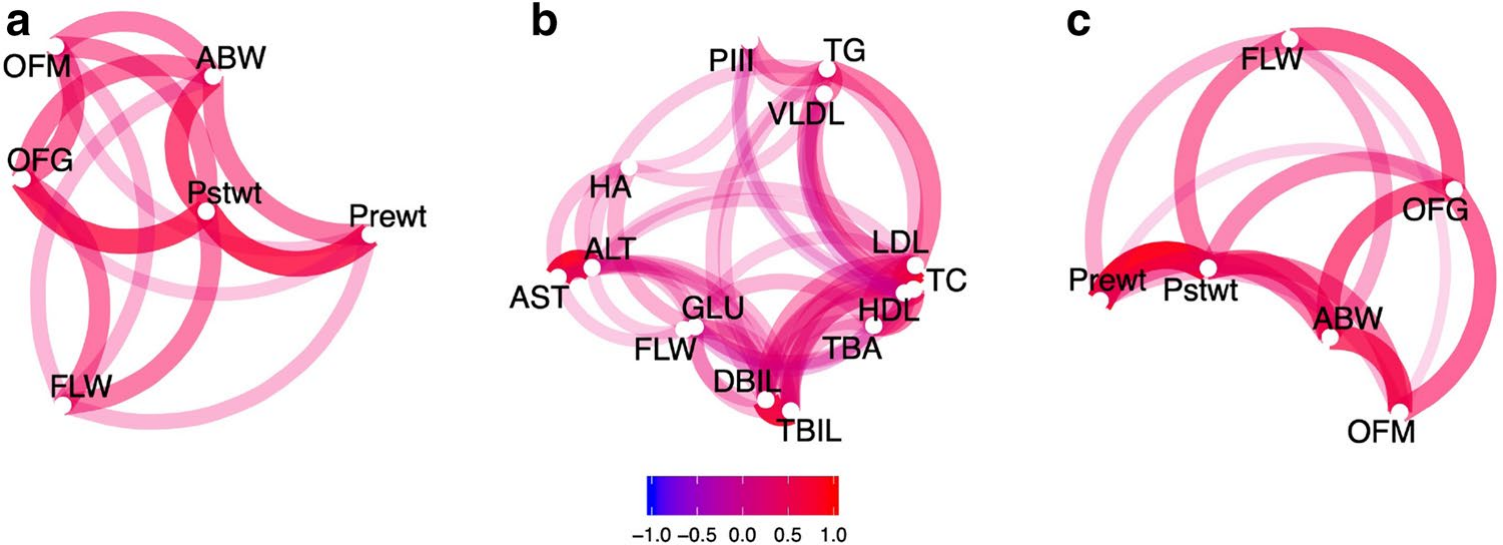
Illustration of the correlations (*r*^2^ > 0.1; *Pearson*) between fatty liver weights (FLW), body weight indices (WIs), and biochemistry indices (BCIs). (**a**) Correlations between FLW and WIs. OFM, number of meals when overfeeding ended; OFG, body weight gain after overfeeding; ABW, abdominal fat weight; AST, aspartate transaminase; ALT, alanine transaminase; DBIL, direct blood bilirubin; TBIL, total blood bilirubin; GLU, blood glucose; TBA, total bile acid; VLDL, very-low-density lipoprotein; HDL, high-density lipoprotein; LDL, low-density lipoprotein; TC, total cholesterol; TG, triglyceride; PIII, N-terminal procollagen III; and HA, hyaluronic acid. (**b**) Correlations between FLW and BCIs. (**c**) Genetic relationships among FLW and Prewt/Pstwt/OFG/OFM/ABW. In (**a**), (**b**) and (**c**), red represents positive correlations and blue represents negative correlations. See more details in Supplementary Table S1/S2 online.

In the current study, 780 geese were initially chosen as candidates for the overfeeding experiments and 488 of all geese were collected according to our overfeeding and sampling protocols (Figure S1). Body weight without overfeeding (75 days of age; Prewt) and with overfeeding (95–104 days of age; Pstwt) for all 488 geese were recorded. After slaughter, fatty liver weight (FLW), abdominal fat weight (ABW) and 12 other biochemical indices (BCIs) were tested. The BCIs included aspartate transaminase (AST), alanine transaminase (ALT), direct blood bilirubin (DBIL), total blood bilirubin (TBIL), blood glucose (GLU), total bile acid (TBA), very-low-density lipoprotein (VLDL), high-density lipoprotein (HDL), low-density lipoprotein (LDL), total cholesterol (TC), triglyceride (TG), N-terminal procollagen III (PIII), and hyaluronic acid (HA). FLW ranged from 359.5 to 1,851.0 g and RLW (ratio of FLW to Pstwt) from 5.3 to 21.3%. The large differences in both FLW and RLW observed between individuals illustrated the variation in ability to store lipids in the liver. As shown in Fig. 1, FLW was significantly correlated with Prewt, Pstwt, OFM (number of meals when overfeeding ended) and OFG (body weight gain after overfeeding) (*p* < 0.001; *Pearson*). Correlations were calculated among weight indices (WIs, including Prewt, Pstwt, OFG, and ABW) and BCIs (Fig. 1; *p* < 0.001; *Pearson*). FLW was generally more closely related to WIs (Fig. 1a, Supplementary Table S1 online) than to BCIs. The two highest correlations were found between FLW and OFG (*r*^2^ = 0.46; *Pearson*) and between FLW and Pstwt (*r*^2^ = 0.45; *Pearson*). For the indices reflecting blood lipid levels, no correlations were found with FLW, although five major indices were tested: VLDL, HDL, LDL, TC, and TG (Fig. 1b). The pairwise clustering of AST-ALT, LDL/HDL-TC, and DBIL-TBIL indicated reliable BCI test results (Fig. 1b). Given the high correlations between FLW and Pstwt, the ratio of FLW to Pstwt was calculated to adjust for possible effects of Pstwt. Genetic correlation (*r*_*g*_) between phenotypes was also calculated (Fig. 1c, Supplementary Table S2 online). *r*_*g*_ between FLW and Pstwt/OFG was much higher than others (0.48 and 0.59, respectively). However, lower *r*_*g*_ was found between FLW and Prewt/OFM (0.26/0.07).

### Genotyping by 2b-RAD (restriction site-associated DNA by IIB restriction endonucleases) and whole-genome resequencing (WGS)

In the 2b-RAD genotyping, the average digested unique tag numbers obtained per individual for the two enzymes used (*BsaXI* and *FaII*) were 411,549 and 460,097, respectively. These numbers were consistent with those from simulations (446,571 and 474,499, respectively). The average sequencing depths for these unique tags were above 35 ×. Up to 82.0% and 92.8%, respectively, of the unique tags for the two enzymes aligned to the goose genome assembly (AnsCyg_PRJNA183603_v1.0). Tags with more than 3 reads were chosen for SNP(single nucleotide polymorphism) calling. Lastly, 46,183 and 26,420 well-genotyped SNPs for *BsaXI* and *FaII*, respectively, were obtained in the 488 geese (using 1 SNP/tag). Reducing the threshold to 2 SNPs/tag led to identification of 80,709 SNPs in total. Four individuals were included as technical replicates in the analysis to evaluate the genotyping accuracy of this method. A concordance of > 90% was found between replicates for > 97.5% of the scaffolds (Supplementary Fig. S2A–D online). For selective sweeps analysis, 24 individuals (14 with very high and 10 with very low blood lipid levels) were selected for whole-genome resequencing on the Illumina X10 platform. In total, ~ 163.5 Gb of raw data were obtained, with more than 96% of the reads aligned to the reference genome. The sequencing depths for the individuals ranged from 8.8 × to 17.0 × , with 96% of the genome covered by high-quality reads. After quality control, 6,007,381 SNPs were identified across 2,699 scaffolds (mitochondria not included). Using the BEAGLE4 software, SNPs from the 80 K genotyped with the 2b-RAD were imputed to higher density facilitated by the whole-genome sequencing. After strict filtration (each marker should have genotyping probability above 0.8 in more than 90% of samples), 480,862 SNPs were kept for the downstream analyses. Further, 3 of the samples analysed using 2b-RAD sequencing were whole-genome re-sequenced to evaluate the imputation quality. For 97.2% of the scaffolds, the concordance of the SNP genotypes was > 90%, indicating high quality for imputed genotypes (see Supplementary Fig. S2E–G).

### Genome-wide association analysis to detect the genetic basis of variation in WIs

**Figure 2:**
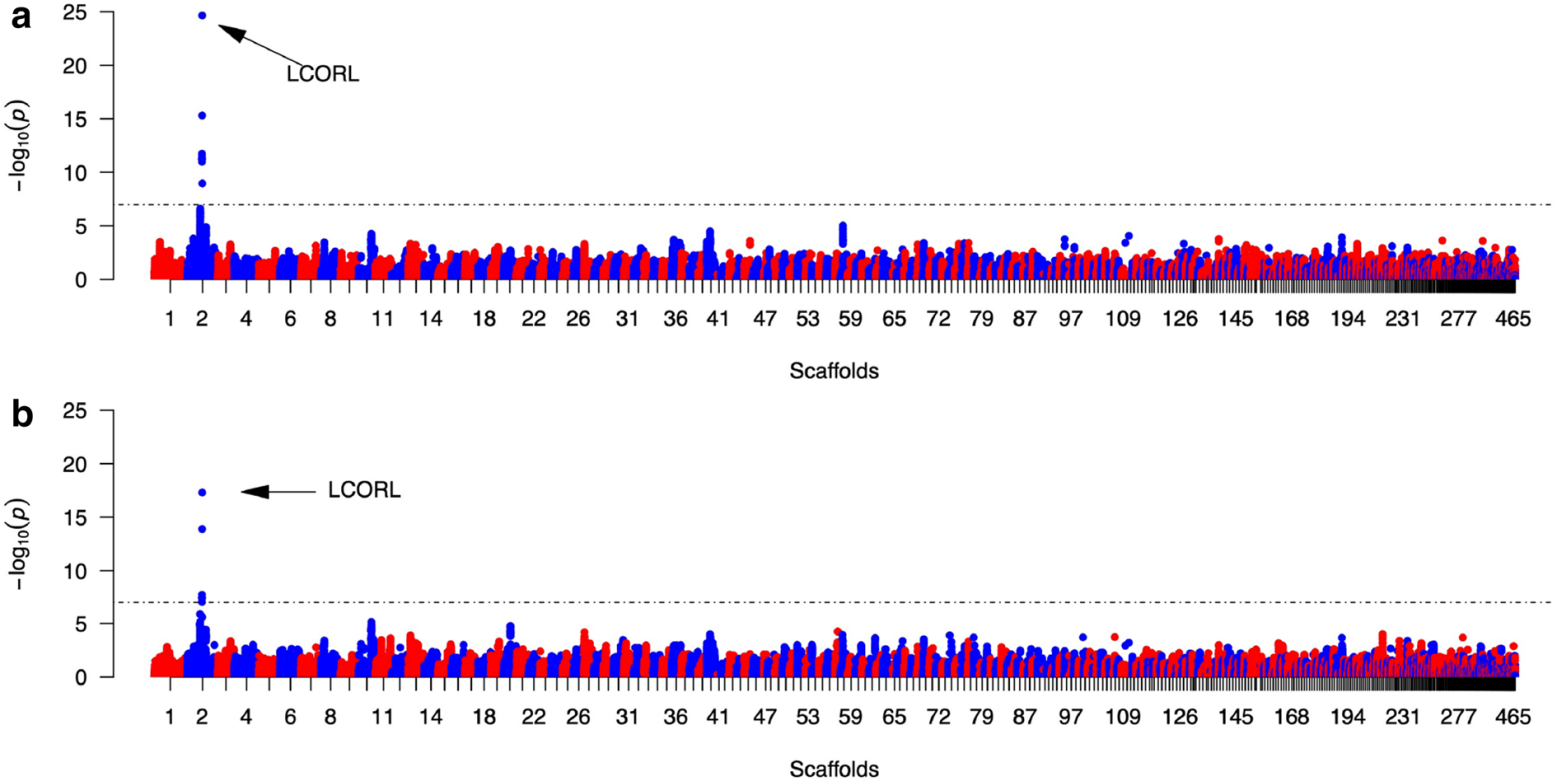
Genome-wide association studies on body weights with (**a**) and without (**b**) overfeeding. Dashed lines in both Manhattan plots refer to significance thresholds at 5% Bonferroni level (− log_10_(0.05/477,243)). The x-axis shows the scaffold numbers assigned by length to make the results more readable (full details on nomenclature are listed in Table S4).

After quality control, 466 samples and 477,243 SNPs were kept in our dataset for genome-wide association studies (GWAS). For each trait within WIs and BCIs, a genome-wide association study was carried out. Except for Prewt and Pstwt, no significant associations were identified, not even at a permissive false discovery rate (FDR) threshold of 0.05 (see Supplementary Fig. S3 and Table S3). Association studies on Prewt and Pstwt detected significant signals and mapped regions were located between 11.6–11.9 Mb (for Prewt) and 11.6–11.8 Mb (for Pstwt) on scaffold 2 (RefSeq: NW_013185655.1, Fig. 2). The variance explained by the most significant marker at 11,846,729 bp on scaffold 2 was calculated, to estimate its effect size. This variant can explain 26.3% and 18.4% of the phenotypic variance in Prewt and Pstwt, respectively. The effects on phenotypes of different genotypes at position 11,846,729 bp and the variance explained by another 10 markers in the region can be found in Fig. S4A–D. Several candidate genes in mapped regions were also found, including *QDPR*, *LAP3*, *LOC106035542*, *MED28*, *FAM184B*, *LOC106035556*, *NCAPG,* and *LCORL*. The two highest signals shared by Prewt and Pstwt were located at either the 5′-end or the 3′-end of the *LCORL* gene. For traits of interest (RLW, FLW, Prewt, and Pstwt), the heritability was estimated to be 0.22, 0.27, 0.48, and 0.31, respectively.

### Genomic regions with large allele-frequency differences between geese with extreme liver weights

**Figure 3:**
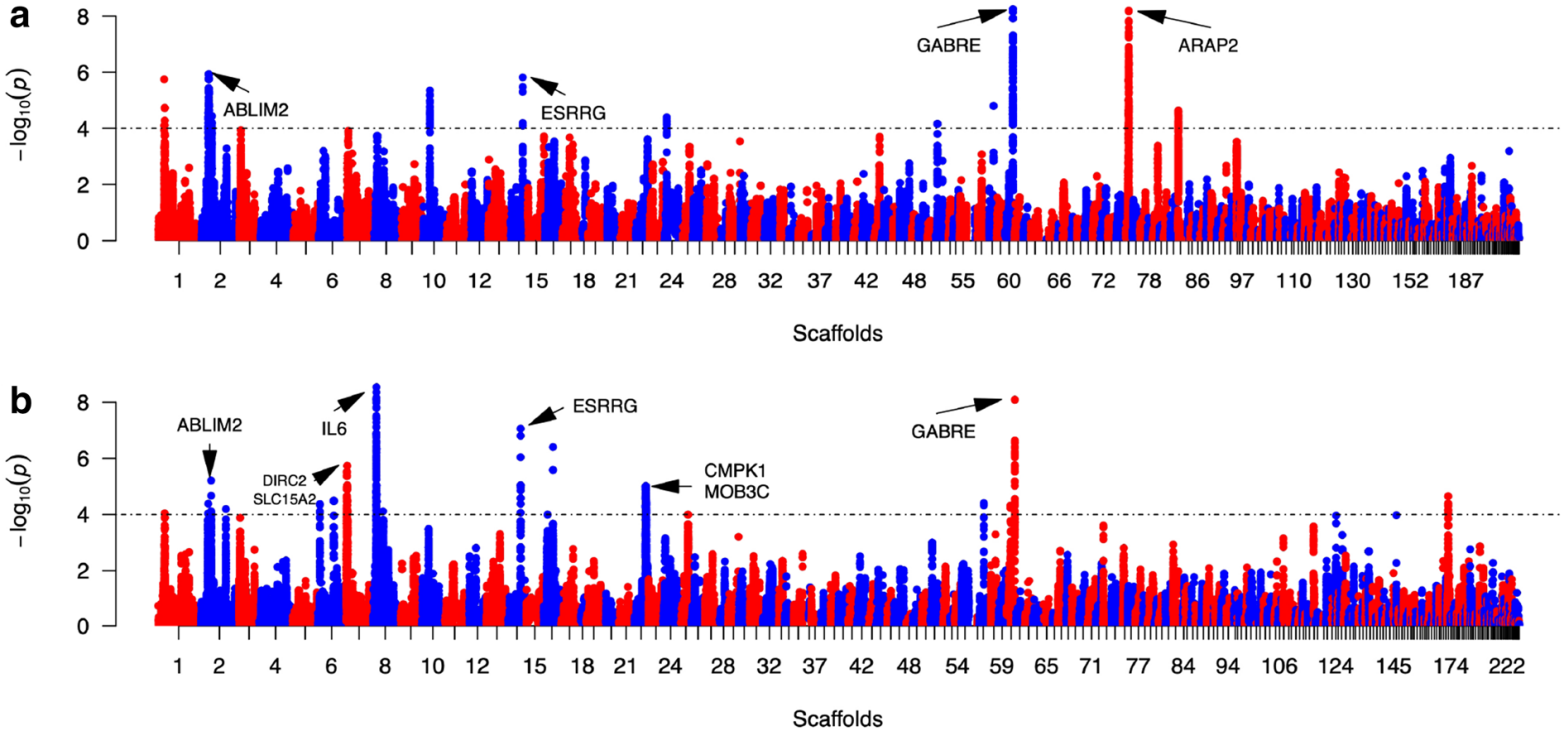
Manhattan plots for the genome-wide ratio of EHHS between populations (Rsb) analysis on overfed geese with divergent ratios of FLW to Pstwt (**a**) and fatty liver weights (**b**). The y-axis shows the transformed *p* values from the Rsb statistics. The significance threshold (dashed line) is set to a log_10_ (two-tailed *p* value) of 4. The x-axis shows the scaffold numbers assigned by length to make the results more readable (full details on nomenclature are listed in Table S4 online).

Using haplotype-sweep methods, multiple regions in the genome were identified where divergent allele-frequencies were found for groups of geese with extreme RLW and FLW (Fig. 3 and Supplementary Table S6). Values for the ratio of EHHS (site-specific extended haplotype homozygosity) between populations (Rsb), between high (1,504.88 ± 98.12 g) and low (757.90 ± 93.88 g) FLW geese, identified significant allelic divergence in regions on scaffolds 1, 2, 6, 7, 8, 14, 16, 22, 58, 61, and 186 (Table S3). In the Rsb analysis between high (0.19 ± 0.01) and low (0.1 ± 0.01) RLW groups, significantly divergent genomic regions were found on scaffolds 1, 2, 10, 14, 24, 52, 59, 61, 76, and 84. The two analyses identified overlapping genomic regions on scaffolds 1, 2, 14, and 61. Within these candidate regions, a total of 25 and 23 genes were identified, respectively (Supplementary Table S3). Signals covering *PANK1*, *HTT*, *ESRRG*, *GABRE,* and *ABLIM2* were detected for both FLW and RLW. *GABRE* is located on scaffold 61 and had the highest signals in the Rsb analysis on RLW. Most variants of *GABRE* were significantly associated with TC, TG, GLU, LDL, VLDL, and the ratio of AST/ALT (*p* < 0.05, Supplementary Table S7). However, no variants within *GABRE* were significantly related to RLW, FLW, or ABW.

In the Rsb analysis of RLW, the highest signals were found in the *ARAP2* gene on scaffold 76. Further, significant associations were detected between polymorphisms in *ARAP2* and FLW, GLU, LDL, as well as VLDL (Supplementary Table S7 online). For both the *GABRE* and *ARAP2* genes, divergent haplotypes with a higher frequency in the groups of geese with low RLW or FLW values were also observed (Supplementary Fig. S5A/B/C online).

In the Rsb analysis of FLW, the most significant signals were found on scaffolds 8 and 61. On scaffold 8, *IL6* was detected with highest signals. Many SNPs (> 25) in or adjacent to *IL6* were associated (*p* < 0.05) with LDL and PIII concentrations (see Supplementary Table S7 online). Divergent haplotypes around *IL6* could also be detected in extremely high- and low-FLW groups (Supplementary Fig. S5D online).In addition, two major facilitator superfamily (MFS) transporter genes on scaffold 7, *SLC15A2* and *DIRC2*, were detected.

### Investigation of lipid accumulation during fatty liver development

**Figure 4:**
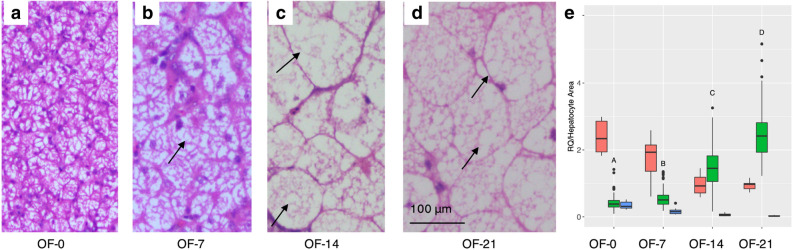
Hepatocyte area of fatty livers sampled at different timepoints and candidate gene expression levels. The black arrows point to lipid droplets in hepatocytes. OF-0/7/14/21 in (**a**–**d**) denote the groups of geese sampled after overfeeding for 0/7/14/21 days, respectively. Figure 4e showed relative expression levels of the *APOB* and *TGH* genes and hepatocyte area (divided by 800). Blue, red and green boxplots refer to *TGH*, *APOB*, and hepatocyte area, respectively. For *APOB* and *TGH*, expression data was stored in Table S5. Different capitalized letters on green box indicated statistical significance for cell areas at different timepoints (*p* < 0.01).

A histology experiment was conducted for all 24 geese to investigate whether the gene expression levels in the non-overfed and overfed groups were related to lipid accumulation in the liver. Significant lipid accumulation was observed in the hepatocytes, especially from OF-7 (overfeed for seven days) to OF-14 (overfeed for 14 days), with very big lipid droplets being present at both OF-14 and OF-21 (overfeed for 21 days, Fig. 4a–d). The most significant change in cell size was from OF-7 (453.3 ± 175.4 μm^2^, *n* = 200) to OF-14 (1,184.2 ± 441.4 μm^2^, *n* = 112). The hepatocyte size grew continuously over time and reached 1,957.3 ± 563.2 μm^2^ (*n* = 128) at OF-21. From OF-0 (no overfed) to OF-21, mass pericellular fibrosis which could be stained blue was not observed. *TGH* and *APOB* genes play critical roles in TG mobilisation and VLDL assembly for TG secretion. In Fig. 4e, gene expression levels of *TGH* as well as *APOB* are seen to be negatively related to hepatocyte area. Hepatocyte area increased significantly over time (*p* < 0.01), while *APOB* and *TGH* were downregulated considerably.

### Genomic regions associated with plasma TG levels

**Figure 5:**
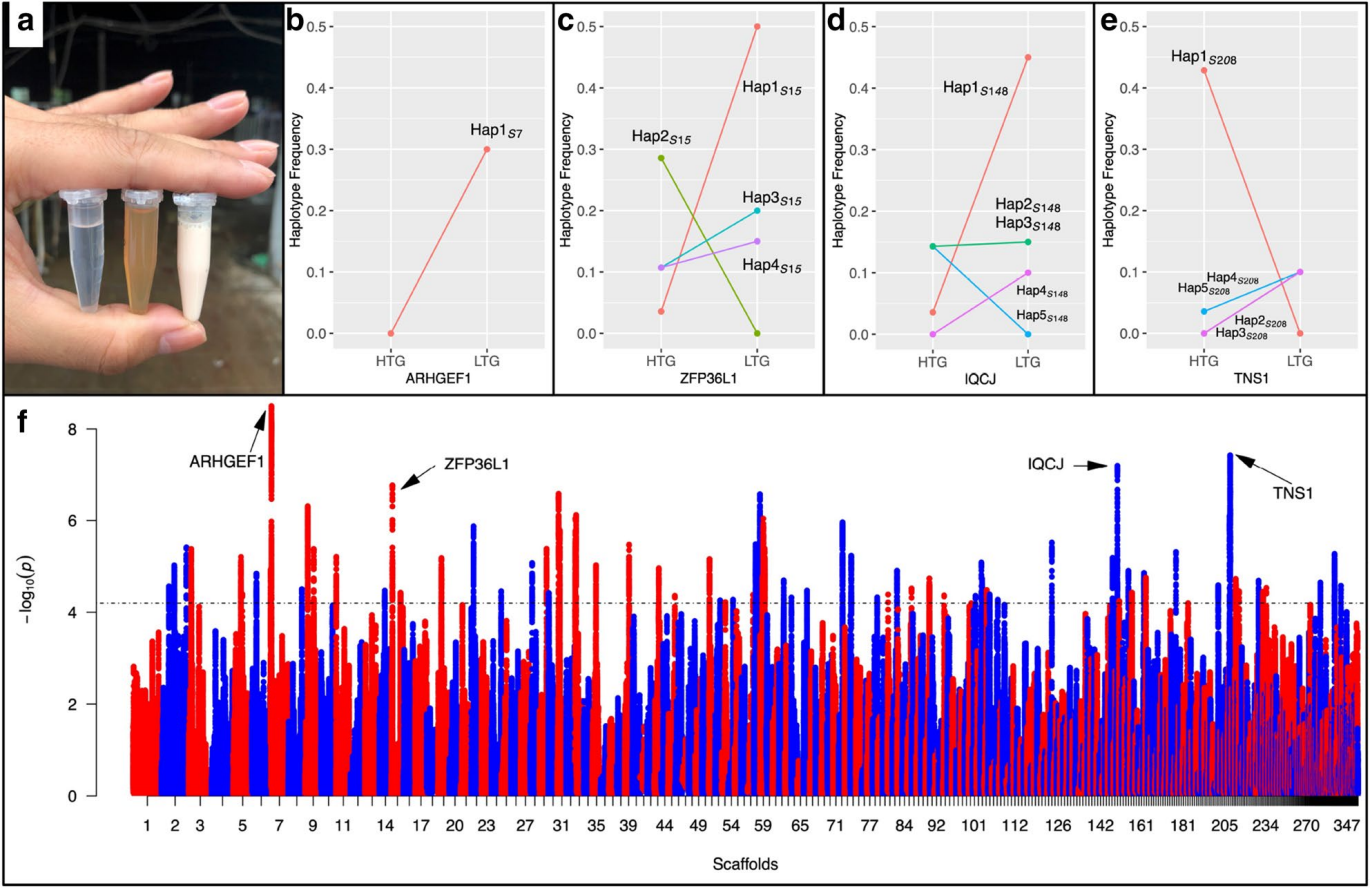
Illustration of the data and results from a haplotype-divergence analysis among geese with different blood TG concentrations. In (**a**), liquids in tubes from left to right were water, serum from overfed geese in the low triglyceride control (LTG) group, and serum from overfed geese in the case group with very high triglyceride (HTG) concentration. (**b**–**e**) show the haplotype frequencies around the *ARHGEF1*, *ZFP36L1*, *IQCJ*, and *TNS1* genes in the HTG and LTG groups. Panel (**f**) shows the Manhattan plot from a selective signature screening between the LTG and HTG groups. The y-axis is − log_10_(*p*) values with the dashed line being the significance threshold (FDR = 0.05). The x-axis is the scaffold numbers as given in Supplementary Table S4.

Plasma TG levels increased quickly from 3.3 ± 1.6 (*n* = 15) mmol/L to 6.2 ± 2.6 (*n* = 287) mmol/L after overfeeding (Supplementary Table S6 online). However, plasma TG levels in 14 samples were much higher (defined as > 20 mmol/L, Supplementary Table S3 online) than those in others, suggesting that these animals were suffering from hyperlipidaemia (Fig. 5a). These samples were used as a case group in a genomic analysis aiming at screening selective signatures. There were 21 phenotypes for individuals in groups with either extremely high or extremely low TG values. The individuals with high TG (*n* = 14) consistently had higher BCIs and WIs, including HA and PIII, which are the two biomarkers for liver fibrosis most commonly used in clinics (Supplementary Table S6 online).

The strongest signals in the haplotype divergence analyses were detected on scaffolds 7, 15, 58, 148, and 208 (Fig. 5f). The region on scaffold 7 contained three genes (*LOC106029886*, *IQCB1,* and *EAF2*) and was close to the region found in the Rsb study on FLW. *LOC106029886* is poorly annotated on the current goose and duck genome assemblies. Given that goose, duck, and chicken have similar gene arrangements across the genome, it is likely that *LOC106029886* refers to *ARHGEF1*. This was further supported by additional protein sequence alignments of *ARHGEF1* from five species (goose, duck, chicken, human, and mouse; Supplementary Fig. S7). One haplotype (Hap1_*S7*_) was only observed in the low TG concentration group (LTG), with a very high frequency (0.3), suggesting its contribution to low blood lipid concentrations (Fig. 5b). Within this gene, SNPs were significantly related to TC, LDL, and TG (*p* < 0.05; Supplementary Table S7). The differentiated region on scaffold 15 contains four genes (*LOC106031663*, *ZFP36L1*, *LOC106031665,* and *HEATR5A*). The strongest signal was located upstream of the *ZFP36L1* gene. One haplotype, Hap1_*S15*_, covering this gene was detected with high frequency (0.50) in the LTG group (Fig. 5c). However, no SNPs were detected in *ZFP36L1*, and no tests for associations between this gene and WIs or BCIs were performed. *IQCJ* on scaffold 148 encodes an IQ motif-containing protein. A SNP at 1,079,381 bp, near *IQCJ*, was significantly associated with plasma TG, LDL, and VLDL concentrations (*p* < 0.05; Table S7). Different dominant haplotypes in LTG and HTG (high TG concentration group) were also found (Hap1_*S148*_ in Fig. 5d). Two peaks were detected on scaffold 208, including one gene *TNS1* (*Tensin 1*) and three long non-coding RNA (lncRNA). Only one SNP was found in *TNS1* gene, at 66,395 bp, and it was significantly associated with ALT (*p* < 0.01) and blood glucose levels (*p* < 0.05). Within this region, one haplotype (Hap1_*S208*_) with differing frequencies in LTG and HTG was also detected (Fig. 5e).

## Discussion

Hepatosteatosis can be found in many wild *Anseriformes,* as a consequence of energy storage before migration. However, susceptibility to hepatic steatosis varies greatly, not only between species, but also between breeds. Within domestic waterfowls, Muscovy duck and Landes goose are more susceptive to steatosis than Peking duck and Poland goose, which are meat-type poultry^19,20^. The difference in capacity to produce fatty liver between breeds results from differing channelling of liver lipids, which means steatosis-sensitive breeds have more retention and less secretion of hepatic lipids than other breeds^19,20^. It was predicted that higher blood lipid levels would correlate with more secreted lipids and less fatty liver production. There might also be negative correlations between blood lipid levels and fatty liver weights. However, our study did not find any significant relationships between fatty liver weight and lipid levels (VLDL, LDL, HDL, or TG), which was surprising. A reasonable explanation for this is the longer overfeeding period (~ 30 days) that was applied in our study, meaning that most geese hit the ceiling of their capacity to store fat. Blood lipid levels have less influence on fatty liver weights in such a long overfeeding period than in a shorter one (14 days)^19,20^. Although blood lipid levels have less direct influence on fatty liver weights, they still play important roles in formation of fatty liver, as we believe resistance to severe hepatosteatosis is a systemic adaptation, which does not occur only in the liver, but in other tissues as well^5^. Within BCIs, AST, ALT, and DBIL were positively related to FLW, even though they do not participate in lipid metabolism. This positive correlation might indicate that the extent of damage owing to hepatosteatosis was proportional to FLW. In contrast to BCIs, WIs appeared to have more effect on FLW, as a closer phenotypic relation was detected. Within WIs, Pstwt and OFG are both phenotypically and genetically correlated with FLW, indicating that genetic influences on the two traits (Pstwt and OFG) have pleiotropic effects on FLW (Fig. 1A, C). Indeed, variants adjacent to *LCORL* which are significantly associated with Pstwt also have significant effects on FLW (Supplementary Fig. S4).

As the cost of high-throughput sequencing and genotyping has decreased, genomic studies have become realistic for many non-model species for which there are no commercial genotyping chips currently available. In our study, high-density markers (> 80 K) from 2b-RAD enabled us to do genome-wide association studies on different traits of interest. This marker set was further imputed to higher density using BEAGLE4, to boost GWAS power in later analyses. In the end, more than 470 K high-quality SNPs were obtained after strict filtration. We attempted to located major genes which might control body weight with or without overfeeding (Pstwt and Prewt) in Landes geese, as Pstwt and Prewt are significantly related to FLW (Fig. 1a). Significant signals for both Prewt and Pstwt were detected and located around the same gene on scaffold 2. The SNP at 11,846,729 bp displayed the most significant association with both Prewt and Pstwt. The phenotypic variance explained by this variant in Prewt and Pstwt was more than 26% and 18%, indicating a “common variants with large effect size” genetic model. In recent years, studies on stature genesis has revealed a simple genetic architecture in livestock animals, in contrast to that in humans, where hundreds of genes all play small roles in controlling body size^21^. The SNP here was located upstream of *LCORL*, which is known as a putative transcription factor that utilises a conserved helix-turn-helix motif for DNA binding^22^ and has been detected as causal gene for body size in independent studies. For example, *LCORL* together with other three genes, could explain more than 80% of body size variation in horse^23^. This is the first time that *LCORL* has been identified as a major gene controlling body size in geese. Our results showed that very few genes have played major roles in the rapid evolution of body size during domestication for both livestock and poultry. Further, the SNP at 11,846,729 bp also had significant effects on FLW, as the FLW of samples with two alternative alleles was significantly greater than that of samples with two reference alleles (Supplementary Fig. S4d).

However, association studies on both RLW and FLW detected no significant signals. We put the eleven significant variants associated with body weight on scaffold 2 into the model as fixed effects, attempting to increase statistical power, but still failed to obtain even a single significant signal. We have two possible explanations for this: (1) association studies indicated a “infinitesimal model” for FLW and RLW, which claimed that many variants are responsible for target traits, but with very small effects. These variants would then explain a very small proportion of the total variance and could not be detected by GWAS. (2) The sample size was too small to detect major genes for RLW and FLW. According to a simulation study, the sample size should be 2,000 if the estimated heritability is around 0.3^24^. We sampled less than 500 geese, which was not enough for detecting potentially causal genes, as the estimated heritability of FLW and RLW was just smaller than 0.3. This also holds true for other traits measured in the current study.

Despite the limited power owing to the small sample and small effect sizes, huge differences in RLW and FLW between samples provided us with the opportunity to conduct a selective signature screening. Using more than 470 K well-genotyped SNPs in a haplotype-based selective sweep analysis among samples with extremely low- and high-RLW or FLW, we identified 48 promising genes (25 in RLW and 23 in FLW). Of these genes, the most important was *ARAP2,* which has been reported as encoding an ADP-ribosylation factor 6 GTPase-activating protein. Knockdown of this gene resulted in decreased lipid droplet formation and TG synthesis^25^. When RNA-Seq data and fat accumulation during fatty liver development were reanalysed, upregulated expression levels of *ARAP2* in the overfed group compared with in the non-overfed group indicated involvement in increasing lipid accumulation (Supplementary Table S8). This suggested to us that the formation mechanism of fatty liver in geese was very similar to that in human although different gene sets were involved. As we know, the most widely identified variant for human NAFLD is located in one lipid droplet remodelling-related genes, *PNPLA3* and this mutation increased fat contents in hepatocytes. Most of the NAFLD-related genes in humans are implicated in lipid droplet remodelling, VLDL secretion, lipogenesis, fibrosis, and innate immunity^7–9,26,27^. This was also seen in our study. Transcriptomic analysis identified two significant GO items related to TG mobilisation and storage (Supplementary Table S9, Fig. S6). Among genes contained in two GO items, lipid storage-promoting genes, including *FITM2*, *PLIN2*, and *DGAT2,* were upregulated, while genes involved in mobilisation of TG stored in lipid droplets and substrate transportation, including *APOB*, *TGH*, *LPIN1*, *SLC15A2*, *DIRC2,* and *SLC37A4,* were downregulated. Indeed, overfeeding significantly decreased the expression levels of LD mobilisation- and secretion-related genes (*APOB* and *TGH*) while increased lipid contents, indicating that altered expression levels of candidate genes had much influence on fat contents. However, more studies on the underlying mechanism how these candidate genes contribute to hepatic fat accumulation are still required in future.

We also found a difference in the fatty liver formation mechanism between goose and human. The goose has evolved to be sensitive to energy intake and has a greater tendency toward steatosis. Once overfed or having access to high-energy feed, geese will develop fatty liver because fat storage-promoting genes (such as *ARAP2*, *FITM,* and *PLIN2*) are susceptive to being upregulated. In humans, however, only people carrying certain mutations in hepatic genes (*PNPLA3*, *PYGO1*, *TM6SF2,* and *HSD17B13*) will develop NAFLD, as these mutations disrupt TG mobilisation and secretion, leading to fat accumulation^28^. Although some genes were not discussed intensively due to ambiguous functions (such as *GABRE* and *ESRRG*), our results provided us with more insights into the genetic architecture shaping the ability of geese to accumulate lipids in such a short time.

According to our histology results, the goose liver stores dramatically increased lipids by enlarging the hepatocyte size, in the same way as human liver does^29^. Hepatocyte hypertrophy increased cell volume to store lipids but could also lead to lipid leakage, if cells failed to keep their shape and structure intact. Lipid leakage is detrimental, as it can result in lipotoxicity followed by inflammation, cirrhosis, and liver failure^30,31^. Two genes, *ABLIM2* and *TNS1*, could be involved in regulating cell morphology when excessive lipids are retained. *ABLIM2* encodes a protein in the ABLIM protein family that serves as a scaffold for signalling modules of the actin cytoskeleton^32^. *TNS1* encodes a focal adhesion molecule that links the actin cytoskeleton to integrins and forms signalling complexes through its multiple binding domains^33^. The much higher frequency of Hap1_*S208*_ in HTG indicated its involvement in higher FLW as samples in HTG had heavier fatty liver than those in LTG.

Steatosis in human could be considered as an upstream causal factor, as it is accompanied by other symptoms of metabolic syndrome, including hypertriglyceridemia and cardiovascular disease^34^. Increased fat content in goose liver was also accompanied by raised serum lipid profiles, including LDL, VLDL, HDL, TC, and TG. Several samples (2.9% of the total) had extremely high lipid levels and seemingly failed to regulate blood lipid concentrations. A rapid and dramatic increase in blood lipid levels could result in many related diseases, such as coronary heart disease^35^ and hypertension^36^. For most geese (more than 97%), plasma TG concentrations were raised, but not to the high levels of samples in the HTG group. We detected three candidate genes, *IQCJ*, *ZFP36L1,* and *ARHGEF1,* which were associated with extremely high blood lipid levels. *IQCJ* (IQ motif-containing J) has been reported to regulate blood TG concentrations through altered expression levels, and SNPs in or near this gene have been found to be highly associated with blood TG levels in human^37–39^. This was confirmed by our results, as more than 14 times higher expression levels of *IQCJ* were detected in the overfed group versus the non-overfed group. *ZFP36L1* (also called *RNF162B*) encodes an RNA-binding protein and is involved in bile acid (BA) metabolism by mediating activation of Cyp7a1 (rate-limit enzyme of BA synthesis)^40^. All seven SNPs located in *ZFP36L1* were significantly related to TBA levels (Supplementary Table S7). Due to its functions in BA metabolism, *ZFP36L1* can also alter lipid absorption in enterohepatic circulation^41^ and significant positive correlation between TBA and FLW was detected (*p* < 0.001). We assume this gene has undergone artificial selection because (1) food for goose wild ancestors contained much less fat and the ability to absorb lipids from intestine would have been enhanced by selection since domestication, as feed contains much higher fat content (5‰), and (2) divergent haplotypes within *ZFP36L1* would have consequences on selection, as HTG with dominating Hap2_*S15*_ would have larger FLW and LTG with dominating Hap1_*S15*_ would have lower FLW (Fig. 5C, Table S6). *ARHGEF1*, RhoA guanine exchange factor 1, plays a key role in salt-induced high blood pressure by mediating the activation of RhoA through the type 1 angiotensin II (Ang II) receptor (*AT1R*), which is essential for Ang II-dependent hypertension in human^42^. This function is realised through the RhoA/Rho-kinase pathway, which also promotes liver fibrosis and portal hypertension by activating hepatic stellate cells^43^. Despite the suggested involvement of these genes in hyperlipidaemia, hypertension, and fibrosis during hepatosteatosis development, the underlying mechanisms of their functions are still not completely clear and require further rigorous experiments. For example, more samples with divergent phenotypes should be collected in future to increase the statistic power in selective sweep analyses and capture more candidate genes.

It was believed that geese are protected from severe pathological damages during formation of fatty liver by mechanisms which remain unknown^18^. This does not mean that geese are immune to hepatosteatosis; we detected increased inflammation and damage levels as hepatic fat content increased, reflected by increased HA, PIII, AST, ALT and DBIL. We assume that the resistance might result from less predisposition toward fibrosis, which would keep hepatosteatosis benign. This is very common in humans; many people are diagnosed with benign NAFLD and can recover without pathogenic damage if they accept treatment. We provided some evidence of how geese prevent hepatosteatosis from worsening and avoid inflammation and fibrosis: (1) Increased damage in goose liver was accompanied by increased protective components, such as superoxide dismutase (SOD)^3^. SOD, encoded by *SOD 1/SOD 2/SOD 3*, which constitutes an antioxidant enzyme defence system against reactive oxygen species (ROS). *SOD2* has been linked to reduced fibrosis in NAFLD patients, indicating that decreased oxidative stress is beneficial for liver damage due to steatosis^44^. *SOD3* was significantly upregulated in the overfed group and might play a similar role in protecting against ROS during fatty liver formation in geese. (2) Although *IL6* (known as inflammation and fibrosis promoting cytokine) was upregulated by 9 times in overfed group, divergent haplotypes of *IL6* genes in high and low FLW groups might indicate that they have differential functions. (3) *ABLIM2* and *TNS1* helped maintain the hepatocyte structure intact and decreased the risk of lipid leakage. As the mechanism of resistance to severe steatosis and predisposition to fibrosis is a critically important scientific matter, more rigid experiments on functional characterisation of candidate genes should be conducted to explore this^18^.

## Conclusions

We found genomic regions containing important candidate genes that differed widely between groups with extreme RLW, FLW, and blood lipids levels. Several of the candidate genes were likely to be involved in modulating hepatocyte size, lipid mobilisation and transportation, and serum lipid concentration. Divergent haplotypes within these genes suggested positive selection on the capacity to store lipids and the ability to protect the liver from damage in an acute phase, even though this was accompanied by increased risk of inflammation and fibrosis. Based on this and results from transcriptomic data, we preliminarily concluded that the mechanism of fatty liver formation in geese is analogous to that in human NAFLD, with both being due to extreme imbalance of lipid synthesis versus catabolism, but involving different sets of genes. These results suggest a systemic evolution in geese to adapt to abnormal lipid metabolism, as liver, circulatory system, and adipose tissue were all involved. Our study detected three genes (*ARHGEF1*, *ZFP36L1,* and *IQCJ*) which are potential targets for treating lipid-related metabolic diseases.

## Methods

All animal protocols were approved by the Shanghai Science and Technology Committee (STCSM) with a license number of SYXK (HU): 2015-0007, and carried out in accordance with approved guidelines and regulations. Every effort was made to minimise the suffering of the geese in the current study.

### Sample and phenotyping

In the current study, 780 geese were initially chosen as candidates for the overfeeding experiments. These geese were hatched on the same day and raised together under identical conditions. At the age of 72 days, all geese were transferred to cages and assigned to four experienced workers in two farm houses. Overfeeding experiments were carried out in November, when the average temperature was approximately 20 °C. There was a three-day pre-overfeeding period for the geese to adapt to the new environment and initiate the metabolic adaptation to overfeeding by giving them the correct diet. The diet used in pre-overfeeding and overfeeding contained nutrition of 2,500 kcal/kg, 80 g/kg crude protein, and 5 g/kg fat. Before the overfeeding experiments, body weight was recorded. During the experiments, all geese were artificially force-fed twice a day on days 1–2, 3 times a day on days 3–10, 4 times a day on days 11–20, and 5 times a day on days 21–30. The amount per meal depended mainly on body size and varied between 250 and 650 g at the same stage of overfeeding experiments. Workers checked each goose every 5–6 h and picked which should be slaughtered. These geese were labelled by painting and deprived of feed for ~ 12 h before being slaughtered using an electrolethaler. Whole blood was sampled and stored unagitated for at least 1 h in two vacuum tubes, one for DNA extraction and another for serum preparation. Goose blood serum samples were stored at − 20 °C for biochemical index tests as soon as the last blood sample was taken. After exsanguination, body weight was measured for each goose. Proper ventilation was always guaranteed in daily management throughout the entire experiment. After removing samples from sick and dead geese, 488 geese were kept for further analyses during the last ten days of overfeeding. See more details in Figure S1A.

### Histology and qPCR experiments

Another overfeeding experiment including 24 geese was performed to both study gene expression profiles and investigate lipid accumulation in the liver at different timepoints during overfeeding (see Supplementary Fig. S1B). Of these 24 geese, six were not overfed and considered as samples at the starting timepoint. The remaining 18 geese were overfed starting at day 75 and their liver tissues were sampled on the 7th (*n* = 6), 14th (*n* = 6), and 21st (*n* = 6) day after initiation of overfeeding. Liver tissues were immersed in 10% formalin buffer for HE (haematoxylin and eosin), for staining performed at Changhai Hospital (Shanghai, China), or in liquid nitrogen for RNA extraction. One HE staining section was prepared for each goose and twenty-four sections were obtained finally. For each section, at least 20 cells were counted to calculated hepatocyte area. Hepatocyte areas were measured using built-in software (cellSens V1.18) in the OLYMPUS microscope.

For qPCR, the total RNA of liver tissues stored in liquid nitrogen was isolated using the TRIzol Reagent (Invitrogen Life Technologies, Carlsbad, CA, USA), in accordance with the manufacturer’s instructions. RNase-free DNase I (Takara, Beijing, China) was used to remove genomic DNA contamination for each sample. The concentration of the RNA was determined using the spectrophotometer ND-1000 (Thermo Fisher Scientific, Wilmington, USA) with the absorbance at 260 nm. The quality of the RNA was detected through 1% agarose gel electrophoresis. First-strand cDNA was synthesised using PrimeScript II 1st Strand cDNA Synthesis Kit (Takara, Beijing, China). Expression profiles of two genes were tested. For *TGH*, the primers (F: 5′-GTTTCTGCTCTTGTCTTATC-3′, R: 5′-TGATGAGGTATAGCATAGC-3′) were designed based on the sequence of MG383670 and amplification product length is 255 base pairs (bp) with optimal *Tm* value between 50 and 56 °C (manuscript in *prep.*). *GAPDH* was used as a house-keeping gene and primers were designed (F: 5′-GGTGGTGCTAAGCGTGTCAT-3′, R: 5′-CCCTCCACAATGCCAAAGTT-3′) and the amplicon length was 200 bp with optimal *Tm* values between 50 and 60 °C. For *APOB*, primers were retrieved from Liu et al*.*^45^. Primers for *TGH*, *APOB,* and *GAPDH* were designed using Primer Premier v5.0 and all three pairs of primers were synthesised at Sangon BioTech (Shanghai, China). qPCR was performed on 7,500 Real-Time PCR systems (Thermo Fisher Scientific, Foster, USA) using the One-Step TB Green PrimeScript RT-PCR Kit (Takara, Beijing, China).

### Biochemical indices (BCIs)

In total, 12 blood chemical indices were measured. AST, ALT, TBIL, TG, TC, DBIL, and TBA were tested using a colorimetric method in an automatic biochemical analyser (BS-420; Mindray Bio-Medical Electronics, Shenzhen, China) using agents supplied by BioSino Bio-Technology and Science Incorporation (Beijing, China). HDL, LDL, VLDL, HA, PIII, and GLU were tested using a radioimmunoassay method with an XH-6020 (Xi'an Nuclear Instrument Factory, Xi’an, China).

### Sequencing, variant calling and imputation

Libraries for 2b-RAD sequencing were constructed for 488 individuals in accordance with the procedures described by Wang et al.^46^, with slight modifications for better implementation. For the allele-frequency divergence analysis, 24 additional samples were used, 14 of which had very high TG levels (> 20 mmol/L) and 10 of which had very low TG levels (< 10 mmol/L). Genomic DNA was extracted from whole blood using QIAprep Spin Miniprep Kit and DNA purified to OD260/280 between 1.8–2.0 for 2b-RAD and whole-genome sequencing (WGS) library preparations. The Illumina HiSeq X10 platform was chosen for resequencing. The Trimmomatic software was used for initial read quality control as described by Bolger et al*.*^47^. The BWA software was used to align quality-controlled data to the reference goose genome (AnsCyg_PRJNA183603_v1.0)^48^, SAMtools was used to transform data formats^49^, and Picard (https://broadinstitute.github.io/picard/) to remove PCR duplications. GATK v3.8 was used for variant calling^50^ and SNP/InDels were annotated using SnpEff^51^. Parameters for filtering variants in the GATK software were: QUAL (phred-scaled probability) < 30, QD (quality by depth) < 2.0, MQ (mapping quality) < 30.0, FS (phred-scaled p-value using Fisher’s exact test) > 60.0, SOR (symmetric odds ratio of 2 × 2 contingency table) > 3.0, MQRankSum (Z-score from Wilcoxon rank sum test of Alt versus Ref read mapping qualities) < 12.5, ReadPosRankSum (Z-score from Wilcoxon rank sum test of Alt versus Ref read position bias) < − 8.0. Only bi-allelic sites with MAF (minor allele frequency) > 0.05 were chosen for subsequent analysis^52^. DNA extraction, library preparation, and sequencing were repeated if sequencing failed due to low-quality raw reads for any sample. In the end, all 488 samples were sequenced successfully with high-quality reads.

Marker density in 2b-RAD experiments was imputed to genome-wide scale using BEAGLE4^53^ based on the more than 6 million markers identified in the resequencing experiments. The software was run with default parameters on each single scaffold and imputed markers were filtered based on estimated genotyping quality above 0.8 in more than 90% of the individuals.

### Genome-wide association studies

For GWAS analysis, SNPs were filtered by setting MAF > 0.05 and Hardy–Weinberg equilibrium (HWE) and call rate > 98% in the GenABEL package^54^. In the end, 3,619 SNPs were removed due to low MAF (< 5%) and 22 samples were excluded due to low call rate. The genome-wide association was performed using a standard mixed model with relationship estimated from genome-wide SNPs to account for population structure and polygenicity. Fixed effects from sex, house and worker were also considered in our model (See Table S10).

1 $${\text{Y}} = {\text{X}}\upbeta + {\text{Zu}} + {\text{e}}$$

Y is the phenotype to be tested (FLW, RLW, Prewt, or Pstwt). X is the design matrix with the number of columns equal to the number of fixed effects plus one, including the worker effect, the goose house effect, and the gender effect, all coded as factors, and the genotype of the SNP tested for association (coded as 0 for minor-allele homozygous and 2 for major-allele homozygous genotypes, respectively). β is a vector of the worker, house, gender, and allelic substitution effects on the corresponding SNP in X. Z is the design matrix obtained from a Cholesky decomposition of the G (identity by state—IBS) kinship matrix estimated from the whole-genome SNP data using the ibs function in the GenABEL package^54^. Z therefore satisfies ${\text{ZZ}}^{\prime } = {\text{G}}$; thus, the random effect vector u will be normally distributed, ${\text{u}}\sim {\text{N}}\left( {0,\upsigma _{{\text{g}}}^{2} } \right)$. e is the normally distributed residual variance with ${\text{e}} \sim {\text{N}}\left( {0 ,\upsigma _{{\text{e}}}^{2} } \right)$. The analyses used the polygenic and mmscore functions in GenABEL package^54^. To estimate the kinship heritability, we fitted a null model by excluding the tested SNP from X, and keeping all the remaining parameters the same. The intra-class correlation r = $\frac{{\upsigma _{{\text{b}}}^{2} }}{{\upsigma _{{\text{b}}}^{2} +\upsigma _{{\text{e}}}^{2} }}$ given by this null model was the amount of variance in y explained by kinship, which is the kinship heritability. In this equation, $\upsigma _{{\text{b}}}^{2}$ refers to additive variance. As the most significant SNP was identified in *LCORL* related to body weight, we also tried to estimate its effects on body weights and fatty liver weight by calculating phenotypic variance explained by this SNP.

2 $${\text{Y}} = {\text{X}}\upbeta + {\text{snp}} + {\text{e}}$$

where Y, X, β and e were same as in formula (1); snp is the markers to be tested.

### Genetic correlations

Weight indices were related to each other significantly and they might share similar genetic background. We used GCTA to perform a bivariate GREML analysis to estimate the genetic correlation between two quantitative traits, including Prewt, Pstwt, ABW, OFG, OFM and FLW^55^.

### Transcriptomic data analyses

RNA-Seq raw data stored under the project numbers PRJNA183603 (Zhedong white goose)^3^ and PRJNA301498 (Landes goose)^18^ were retrieved using the SRA Toolkit (Version 2.10.0) from NCBI. NGS QC TOOLKIT v2.3.2 (https://59.163.192.90:8080/ngsqctoolkit/) was used to trim reads with low quality. Reads with quality < Q20 and length < 35 bp were removed. TopHat was used to align clean reads to a goose genome assembly (− r 50 − *p* 30). The files returned by TopHat stored mapping information which was used by Cufflinks to assemble transcripts (library-type fr-unstranded—*p* 10). Htseq was selected to obtain the number of reads mapped to genes and calculate FPKM values. Landes geese have biological repeats at different timepoints and the function nbinomTest in DEseq was used to estimate differentiated expression levels among groups based on basemean values. Zhedong White geese do not have repeats and the function DGEList from edgeR was used to estimate expression differences between groups. DEGs found in the last step were aligned to Swissprot database to acquire GO items, as geese currently have no annotations for GO items. A basic function called phyper in R was used to calculate P values. The GO terms generally describe our knowledge of the biological domain with respect to molecular functions (MF), cellular components (CC), and biological process (BP). The top ten terms with P values and enrichment score are listed in Supplementary Table S9. In contrast to *APOB* and *TGH* genes which were quantified by qPCR, expression profiles of all other genes in our study (e.g. *IL6*, *ARAP2*, *DIRC2*, *PLIN2*, *ARHGEF1*, *ZFP36L1*) were determined in this step.

### Selective signature scanning among samples with extremely high- and low-FLW/RLW/TG

Rsb (ratio of site-specific haplotype homozygosity between populations) analyses were conducted using the rehh package comparing groups of geese with extremely high and low values of RLW or FLW^56^. To select samples with extreme FLW and RLW, 305 of 488 geese which were kept in the same farm house and had similar body weight were selected as sampling pool. For FLW, 25 and 24 samples with extremely high and low phenotypes, respectively, were selected. For RLW, 22 and 21 samples with extremely high and low phenotypes, respectively, were selected. In these sets of samples for the two different studies, 31 samples shared. Details about the phenotypes in each group can be found in Table S6. The integrated EHHS (site-specific haplotype homozygosity) for each SNP in each population (iES) was calculated, and the Rsb statistics between populations were defined as the natural log of the ratio between populations. In both Rsb analyses, a *p* value = 0.0001 was used as the threshold to select significant Rsb values and genomic regions. All individuals were phased using BEAGLE4 before Rsb analyses.

Throughout our overfeeding experiments, 14 samples were detected with very high lipid levels (4 on days 1–20 and 10 on days 21–30) and 10 with extremely low lipid levels in day 21–30 (see Figure S1). For comparisons between groups with high and low plasma TG levels, the hapFLK software was used to find signatures associated with TG levels. The most important parameter in hapFLK is the cluster value for ancestral haplotypes and this was set to K = 6. P values were estimated from standardised hapFLK values using the rlm function of the MASS package in R, as described by Fariello et al*.*^57^. Based on the approach of Benjamini and Hochberg (1995)^58^, the FDR for using *p* < 10^–4^ was 5%. As the candidate genomic regions with significant signals were detected, haplotypes within these regions were constructed and their frequency was calculated. Sample phenotypes and scripts used in current study were deposited in a GitHub repository (https://github.com/tuzixuexi/Geese_Fatty_Liver).

## Supplementary information

Supplementary Information.

## Data Availability

For the current study, 2b-RAD sequencing data and resequencing data are available in NCBI with project numbers of PRJNA631863, PRJNA575114, and PRJNA575503. Phenotypes and scripts were deposited in a GitHub repository (https://github.com/tuzixuexi/Geese_Fatty_Liver).
