# Supplementary material for: Exploring the genetic basis of fatty liver development in geese
Source: Sci Rep. 2020 Aug 31;10:14279. doi: 10.1038/s41598-020-71210-8 (PMC7459336; doi:10.1038/s41598-020-71210-8)
Supplement: Supplementary file 1 — Supplementary Information. [file 41598_2020_71210_MOESM1_ESM.pdf]

Exploring the genetic basis of fatty liver development in Geese

Yunzhou Yang[1,2*], Huiying Wang[1], Guangquan Li[1], Yi Liu[1], Cui Wang[1], Daqian He[1*]

[1]Institute of Animal Husbandry & Veterinary Science, Shanghai Academy of Agricultural Sciences, Shanghai, 201106, P.R. China

[2]Department of Medical Biochemistry and Microbiology, Uppsala University, Uppsala, 75123, Sweden

[*]Corresponding author:

Daqian He
Institute of Animal Husbandry & Veterinary Science
Shanghai Academy of Agricultural Sciences
201106, Minhang District
Shanghai, PR China
Tel: +86-021-62205473
daqianhe@aliyun.com

Yunzhou Yang
Institute of Animal Husbandry & Veterinary Science
Shanghai Academy of Agricultural Sciences
201106, Minhang District
Shanghai, P.R. China
Tel: +86-021-62205473
Department of Medical Biochemistry and Microbiology
Uppsala University
75 123, Husargatan 3
Uppsala, Sweden
Tel: +46–0790111308
yang.yunzhou@imbim.uu.se

Supplementary Figures

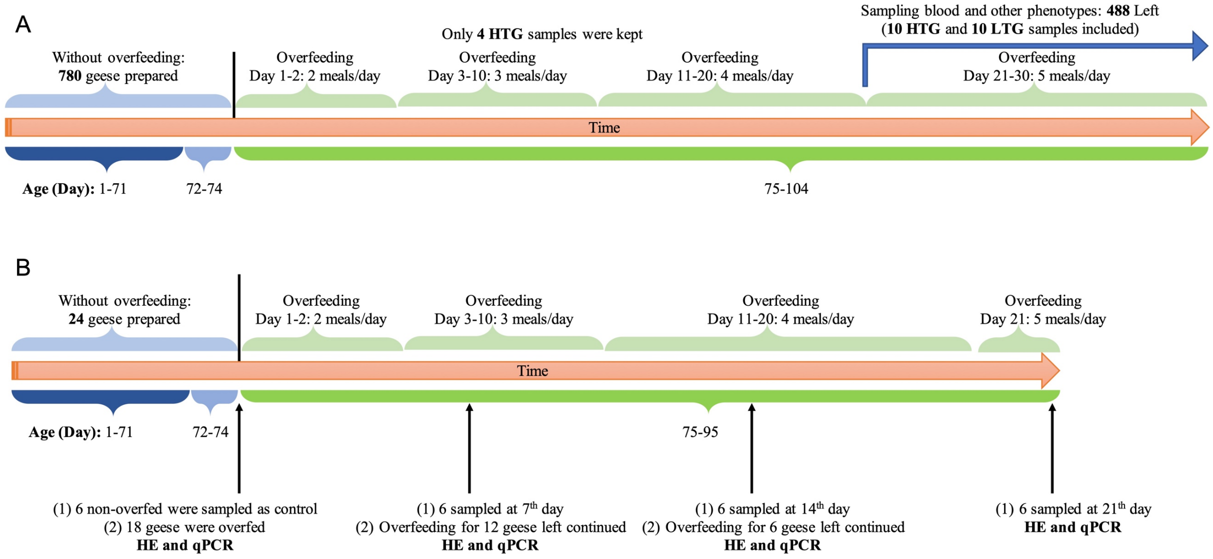

Figure S1 Experiment design. Two experiments were carried out in our study. In Figure A, all 780 geese were initially selected for overfeeding experiments. After 30 days overfeeding, 488 geese were kept for final analysis. Weight indices (WIs) and biochemistry indices (BCIs) were all recorded and tested. Samples with extremely high- (14) and low-TG (10) were also phenotyped. In Figure B, 24 geese were overfed for 3 weeks (one group was overfed) to do HE staining and qPCR. At each time point, we have 6 samples as biological replicates.

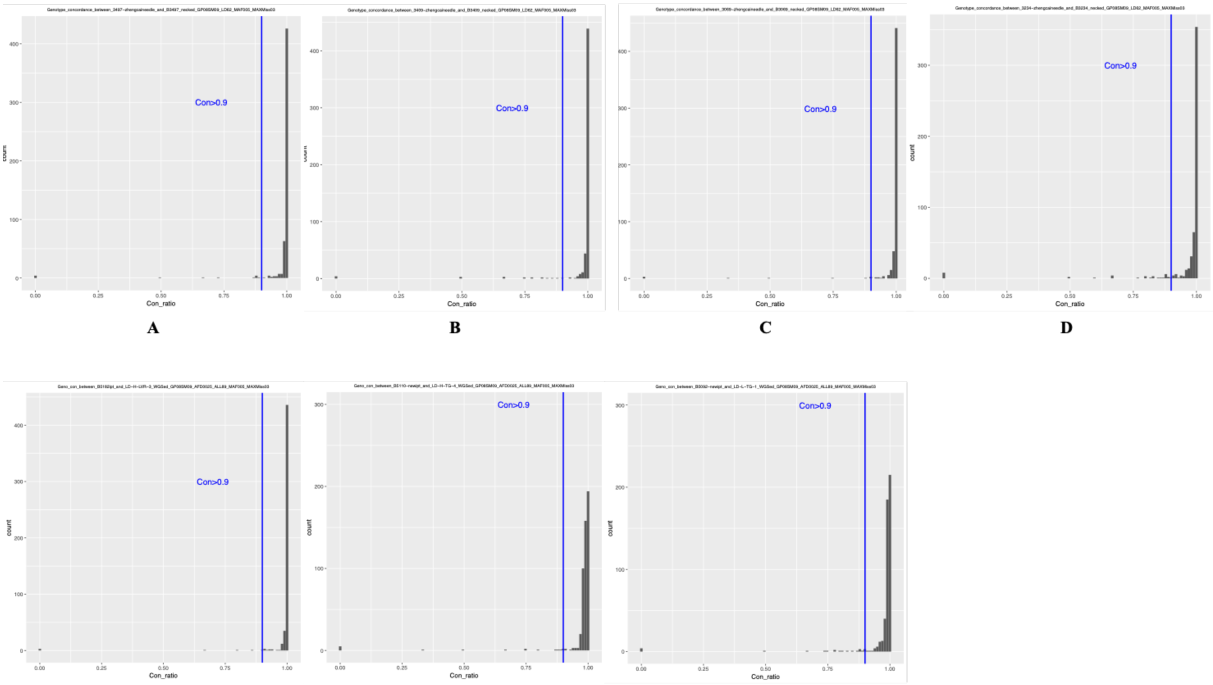

Figure S2 Genotype concordance between repeated samples for 2bRAD methods. Figures A - D: concordance between replicated samples in 2b-RAD experiments. Figures E - F: concordance between imputed and re-sequenced samples. Blue vertical lines indicated 90% concordance threshold.

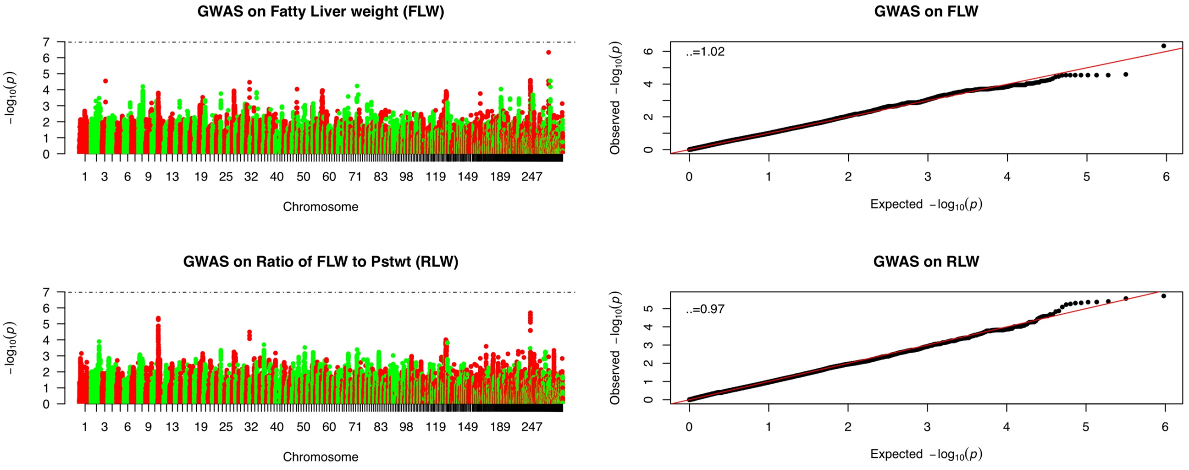

Figure S3 GWAS on RLW (ratio of fatty liver to body weight after overfeeding) and FLW (fatty liver weight). Numbers shown in Q-Q plots were inflation arguments which indicated much less population stratification confounders. Also, scaffold numbers here are defined for more readable information and details could be found in Table S2.

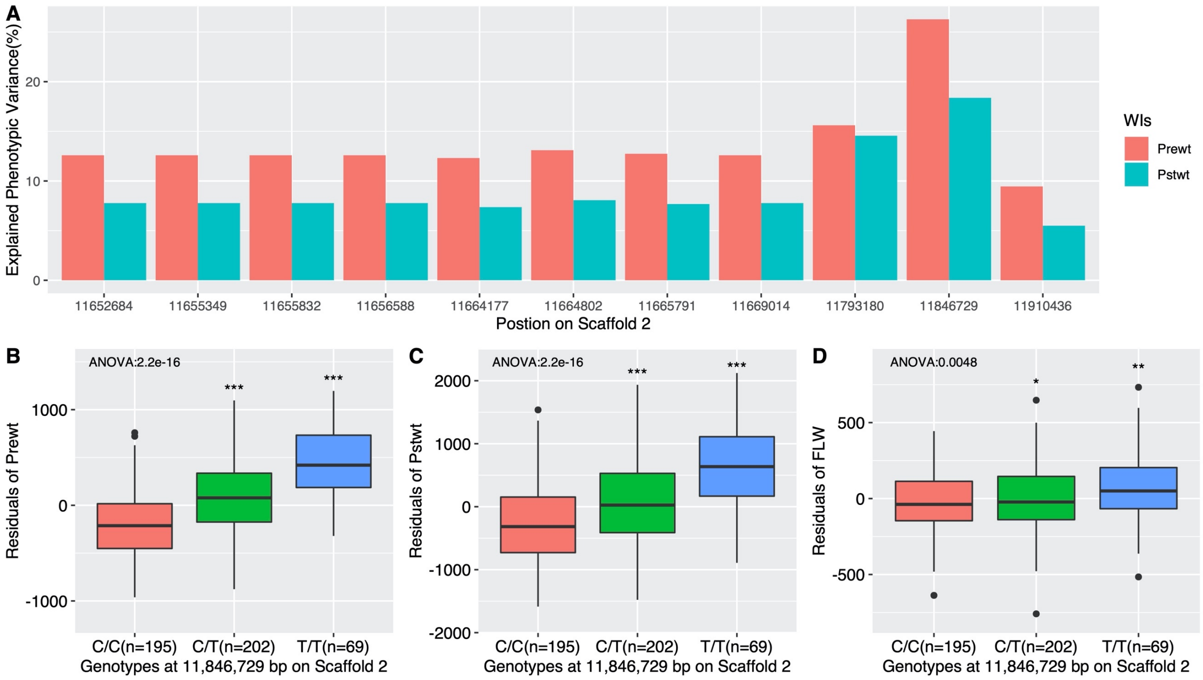

Figure S4 Explained phenotypic variance by all 11 SNPs on scaffold 2 in both Prewt and Pstwet. A: explained variance along the positions on scaffold 2 (NW_013185655.1) in Prewt (red) and Pstwt (green). B – D are phenotypic residuals by fitting sex, worker and overfeeding method with Prewt and Pstwt for three different genotypes at 11,846,729.

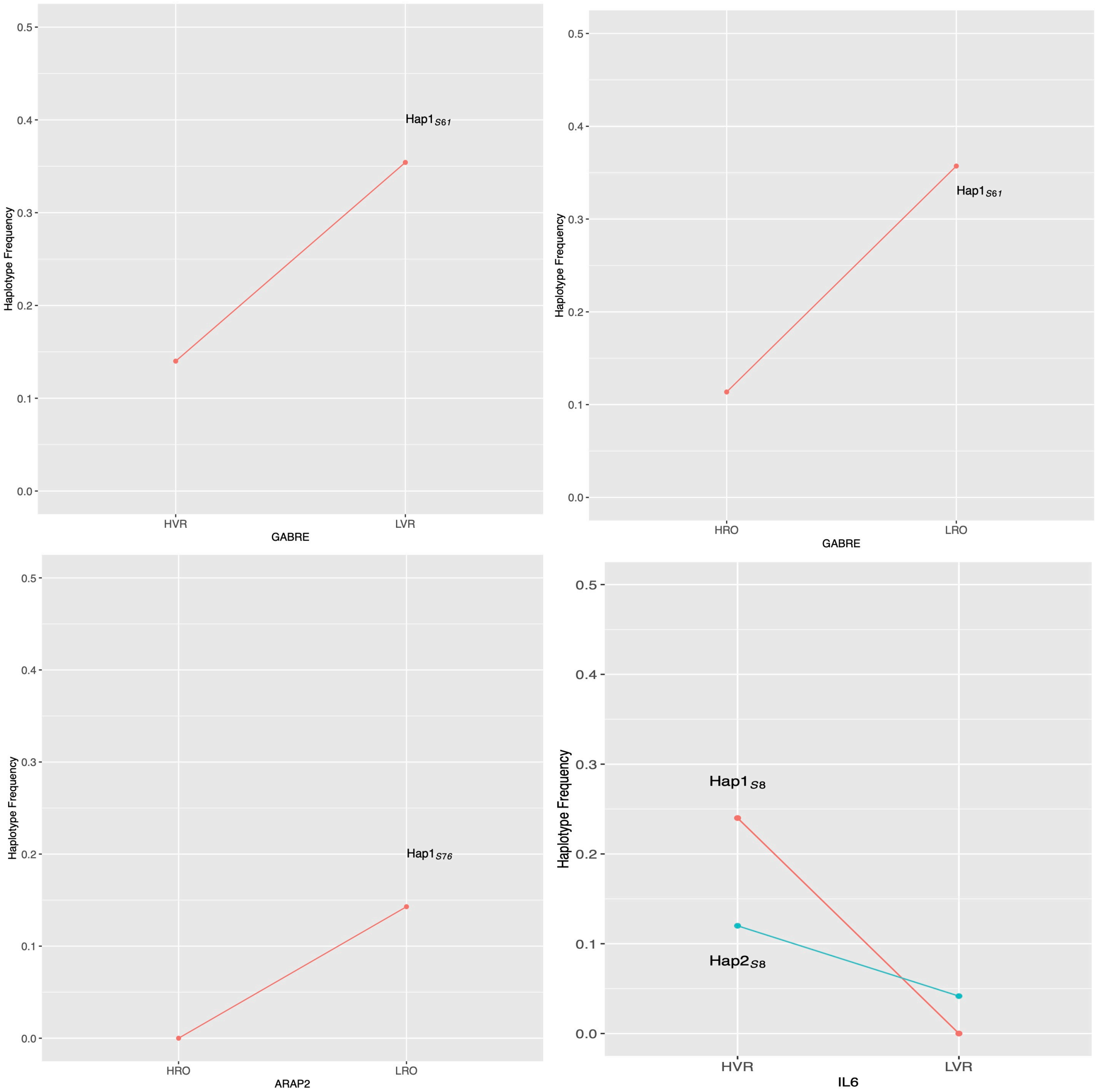

Figure S5 Haplotypes Frequencies around GABRE, ARAP2 and IL6. A: Frequency of haplotypes around GABRE between High- and Low-FLW. B: Frequency of haplotypes around GABRE between High- and Low-RLW. C: Frequency of haplotypes around ARAP2 between High- and Low-RLW, D: Frequency of haplotypes around IL6 between High- and Low-FLW.

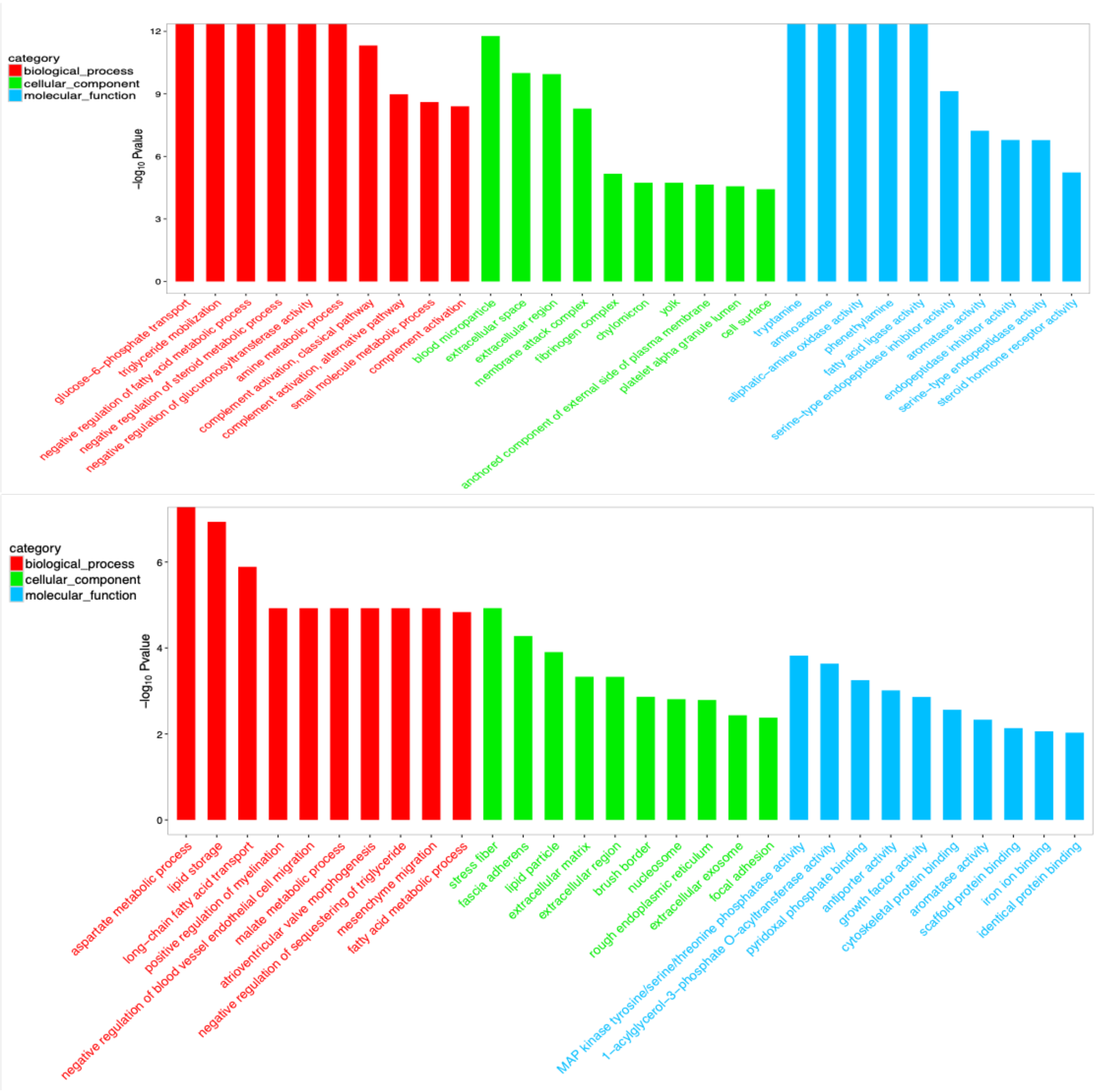

Figure S6 Top 10 GO terms with down or up regulated genes. Above: GO terms in Landes geese. Below: GO terms in ZheDong White geese.

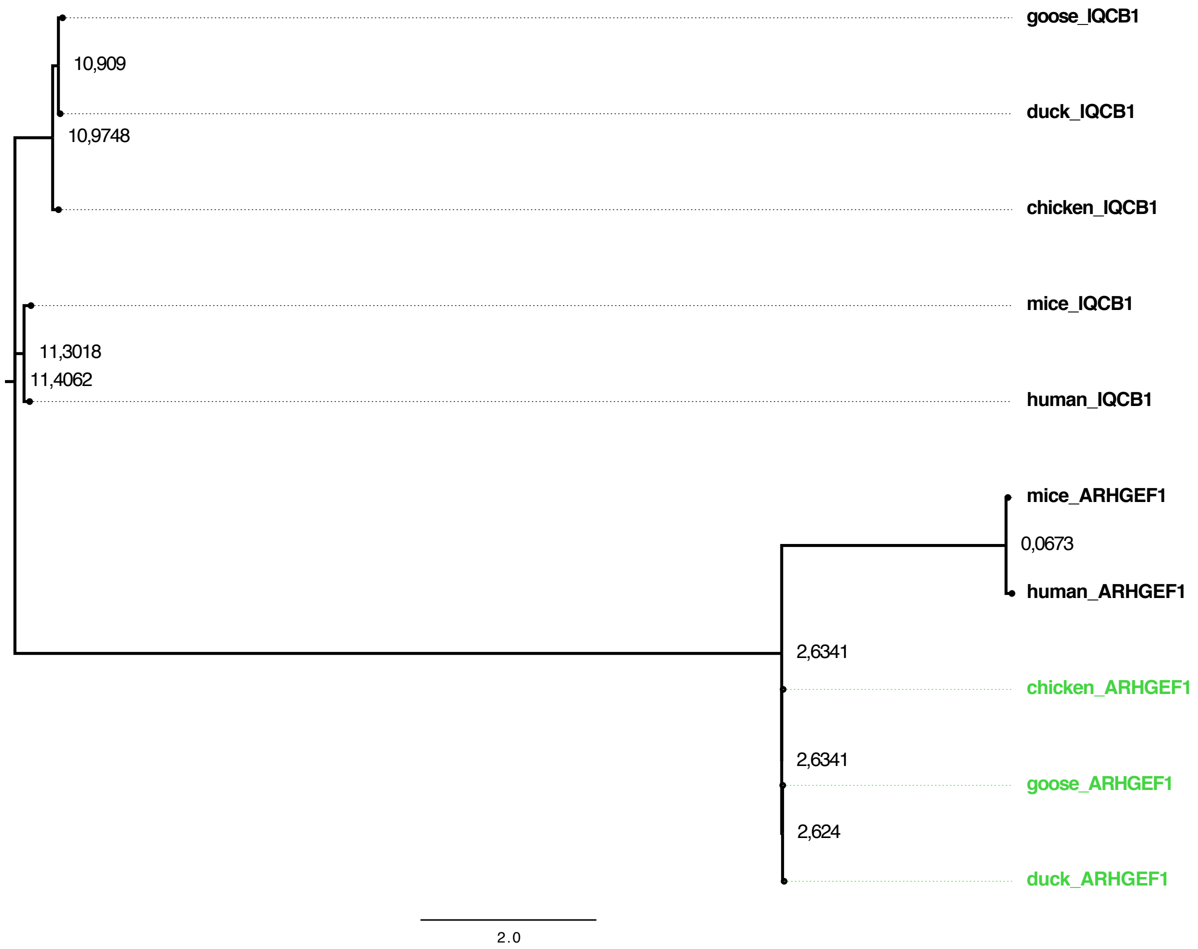

Figure S7 Phylogeny tree based on maximum likelihood algorithms. The tree was constructed by MEGA7 software[1] with 1000 bootstrap values. The figure was plotted by Figtree 1.4.4[2]. Protein sequences of IQCB1 and ARHGEF1 gene in goose. chicken. duck. human and mice were downloaded from NCBI. Accession number for IQCB1 gene: human- NP_001306036.1. mice- XP_006522312.1. chicken- XP_422091.4. duck-XP_027318051.1. goose- XP_013026575.1. Accession number for ARHGEF1 gene: human-NP_004697.2. mice-NP_001123623.1. chicken-XP_025008345.1. duck-XP_027318050.1. goose-XP_013026666.1.

Supplementary Tables

Table S1 Correlations among different phenotypes. Upper triangle is the *Pearson* correlations. lower triangle is the p values.

WIs/BCIs*	Prewt	OFM	Pstwt	OFG	FLW	ABW	RLW	AST	ALT	TBIL	DBIL	TC	TG	HDL	TBA	GLU	LDL	VLDL	PIII	HA
Prewt	1.00	0.08	0.68	-0.08	0.15	0.28	-0.15	0.00	0.01	0.12	0.02	0.03	-0.03	0.05	0.20	-0.07	-0.02	-0.04	0.02	-0.08
OFM	1.00	1.00	0.49	0.59	0.24	0.47	0.05	-0.11	-0.05	0.13	0.11	0.21	0.22	0.05	0.15	-0.06	0.26	0.11	0.10	-0.02
Pstwt	0.00	0.00	1.00	0.68	0.45	0.50	0.04	-0.03	0.00	0.10	0.08	0.08	0.12	0.05	0.23	-0.09	0.05	0.02	0.04	-0.06
OFG	1.00	0.00	0.00	1.00	0.46	0.41	0.21	-0.04	-0.01	0.02	0.09	0.08	0.19	0.02	0.12	-0.06	0.09	0.07	0.04	0.00
FLW	1.00	0.07	0.00	0.00	1.00	0.14	0.91	0.08	0.07	0.29	0.41	0.05	0.15	-0.06	0.28	-0.12	0.06	0.04	-0.05	0.02
ABW	0.00	0.00	0.00	0.00	1.00	1.00	-0.08	-0.12	-0.07	0.07	-0.01	0.14	0.13	0.12	0.15	-0.04	0.16	0.04	0.04	-0.13
RLW	1.00	1.00	1.00	0.64	0.00	1.00	1.00	0.10	0.08	0.27	0.41	0.01	0.12	-0.09	0.20	-0.11	0.05	0.04	-0.07	0.04
AST	1.00	1.00	1.00	1.00	1.00	1.00	1.00	1.00	0.95	0.13	0.16	0.03	-0.01	-0.01	-0.01	0.19	0.02	-0.01	-0.06	0.24
ALT	1.00	1.00	1.00	1.00	1.00	1.00	1.00	0.00	1.00	0.19	0.22	0.13	0.04	0.09	0.06	0.22	0.11	0.03	-0.06	0.25
TBIL	1.00	1.00	1.00	1.00	0.00	1.00	0.01	1.00	1.00	1.00	0.87	0.41	0.15	0.27	0.26	0.28	0.41	0.08	-0.09	0.05
DBIL	1.00	1.00	1.00	1.00	0.00	1.00	0.00	1.00	0.34	0.00	1.00	0.38	0.20	0.21	0.23	0.26	0.39	0.09	-0.03	0.09
TC	1.00	0.51	1.00	1.00	1.00	1.00	1.00	1.00	1.00	0.00	0.00	1.00	0.31	0.78	0.50	0.28	0.91	0.09	0.04	0.10
TG	1.00	0.30	1.00	1.00	1.00	1.00	1.00	1.00	1.00	1.00	1.00	0.00	1.00	-0.04	0.23	0.05	0.38	0.40	0.52	0.10
HDL	1.00	1.00	1.00	1.00	1.00	1.00	1.00	1.00	1.00	0.01	0.43	0.00	1.00	1.00	0.44	0.22	0.61	-0.24	-0.15	0.02
TBA	1.00	1.00	0.11	1.00	0.00	1.00	0.82	1.00	1.00	0.01	0.15	0.00	0.15	0.00	1.00	-0.21	0.45	0.15	-0.07	-0.10
GLU	1.00	1.00	1.00	1.00	1.00	1.00	1.00	1.00	0.39	0.00	0.02	0.00	1.00	0.27	0.73	1.00	0.20	-0.07	-0.03	0.36
LDL	1.00	0.02	1.00	1.00	1.00	1.00	1.00	1.00	1.00	0.00	0.00	0.00	0.00	0.00	0.00	1.00	1.00	0.22	0.11	0.06
VLDL	1.00	1.00	1.00	1.00	1.00	1.00	1.00	1.00	1.00	1.00	1.00	1.00	0.00	0.09	1.00	1.00	0.30	1.00	0.10	-0.05
PIII	1.00	1.00	1.00	1.00	1.00	1.00	1.00	1.00	1.00	1.00	1.00	1.00	0.00	1.00	1.00	1.00	1.00	1.00	1.00	0.20
HA	1.00	1.00	1.00	1.00	1.00	1.00	1.00	0.08	0.04	1.00	1.00	1.00	1.00	1.00	1.00	0.00	1.00	1.00	1.00	1.00

* WIs and BCIs are abbreviations for weight indices (including body weight before overfeeding-Prewt. body weight after overfeeding-Pstwt. fatty liver weight-FLW. body weight gain after overfeeding-OFG. abdominal fat weight after overfeeding-ABW) and biochemical indices. RLW is ratio of FLW to Pstwt. AST (Aspartate transaminase). ALT (Alanine transaminase). DBIL (Direct blood bilirubin). TBIL (Direct blood bilirubin). GLU (Blood glucose). TBA (Total bile acid). VLDL (Very low-density lipoprotein). HDL (High density lipoprotein). LDL (Low density lipoprotein). TC (Total cholesterol). TG (Triglyceride). PIII (N-terminal procollagen III) and HA (Hyaluronic acid).

Table S2 Genetic correlations among weight indices

WIs*	Prewt	Pstwt	FLW	ABW	OFG	RLW	OFM
Prewt	1	0.94	0.26	0.45	0.09	-0.33	0.23
Pstwt	0.94	1	0.48	0.65	0.41	-0.13	0.47
FLW	0.26	0.48	1	0.28	0.59	0.81	0.07
ABW	0.45	0.65	0.28	1	0.61	-0.13	0.7
OFG	0.09	0.41	0.59	0.61	1	0.4	0.55
RLW	-0.33	-0.13	0.81	-0.13	0.4	1	-0.23
OFM	0.23	0.47	0.07	0.7	0.55	-0.23	1

* WIs refer to weight indices, including body weight without/with overfeeding (Prewt and Pstwt), fatty liver weight (FLW), abdominal fat weight (ABW), body weight gain (OFG), ratio of FLW in Pstwt (RLW). OFM refers to number of meals when overfeeding was ended.

Table S3 Candidate genomic regions associated with RLW FLW and Blood lipid

Scaffold	Scaffold	Scaffold Num	Start	End	Genes	Detected by
2	NW_013185655.1	45	17518072	17558 550	VRK1	GWAS
11	NW_013185664.1	61	970437	1029601	MYT1L	GWAS
72	NW_013185725.1	36	618053	618053	BAZ1A	GWAS
128	NW_013185781.1	109	573320	1 975438	OSBPL5. MOB2	GWAS
243	NW_013185896.1	267	781442	781442	MDGA2	GWAS
244	NW_013185897.1	382	210772	277773	MARK3. EIF5. LOC106047103	GWAS
301	NW_013185954.1	129	348330	348330	ADAMTSL1	GWAS
**Scaffold**	**Scaffold**	**Scaffold Num**	**Start**	**End**	**Genes**	**Detected by**
1	NW_013185654.1	65	4317421	4317421	NA	Rsb on FLW
2	NW_013185655.1	45	5036899	5039122	HTT	Rsb on FLW
			6504697	6516128	ABLIM2	Rsb on FLW
			14478731	14530100	SEL1L3	Rsb on FLW
6	NW_013185659.1	88	1242836	1246345	LOC106029463	Rsb on FLW
			8665976	8674477	LOC106029463	Rsb on FLW
7	NW_013185660.1	23	1056450	1159385	DIRC2	Rsb on FLW

			971940	1039488	SLC15A2	Rsb on FLW
			1060969	1087402	HSPBAP1	Rsb on FLW
			1107225	1111134	LOC106029891	Rsb on FLW
			1286378	1319364	SEMA5B	Rsb on FLW
8	NW_013185661.1	24	2374227	2391179	LOC106030192	Rsb on FLW
			2475532	2571095	LOC106030195	Rsb on FLW
			2475532	2571095	LOC106030127	Rsb on FLW
			2475532	2571095	LOC106030196	Rsb on FLW
			2475532	2571095	IL6	Rsb on FLW
			6024649	6024649	GALNT15	Rsb on FLW
14	NW_013185667.1	58	7032568	7060961	ESRRG	Rsb on FLW
16	NW_013185669.1	20	5227000	5229589	SORCS3	Rsb on FLW
22	NW_013185675.1	26	6820283	6845885	CMPK1	Rsb on FLW
			6972839	6984552	MOB3C	Rsb on FLW
58	NW_013185711.1	55	3043972	3047147	MAP7D3	Rsb on FLW
61	NW_013185714.1	93	2366851	2372113	AFF2(FMR2)	Rsb on FLW
			4647533	4700148	GABRE	Rsb on FLW
186	NW_013185839.1	107	920242	925390	CCDC37	Rsb on FLW
**Scaffold**	**Scaffold**	**Scaffold_Num**	**Start**	**End**	**Genes**	**Detected by**
1	NW_013185654.1	65	4039550	4045170	**PANK1**	Rsb on RLW
			4317421	4340217	LOC106033540	Rsb on RLW
2	NW_013185655.1	45	4680402	4691368	TNIP2	Rsb on RLW
			4824117	4857279	ADD1	Rsb on RLW
			4857493	4883254	MFSD10	Rsb on RLW
			4889010	4912343	NOP14	Rsb on RLW
			4954016	5039122	HTT	Rsb on RLW
			6508971	6526979	ABLIM2	Rsb on RLW
10	NW_013185663.1	37	4172985	4187961	LOC106030633	Rsb on RLW
14	NW_013185667.1	58	7032568	7060961	ESRRG	Rsb on RLW

24	NW_013185677.1	273	1182957	1193704	CLUAP1	Rsb on RLW
52	NW_013185705.1	81	1241701	1242015	NA	Rsb on RLW
59	NW_013185712.1	40	4335439	4335487	NA	Rsb on RLW
61	NW_013185714.1	93	4613693	4627217	GABRA3	Rsb on RLW
			4630697	4677071	GABRE	Rsb on RLW
76	NW_013185729.1	170	2872215	2880884	LOC106039086	Rsb on RLW
			2904318	2989485	ARAP2	Rsb on RLW
84	NW_013185737.1	158	1123381	1129773	DLG2	Rsb on RLW
			1129992	1135514	TMEM126A	Rsb on RLW
			1139315	1142902	CCDC89	Rsb on RLW
			1145075	1153535	SYTL2	Rsb on RLW
			1143387	1144994	LOC106039888	Rsb on RLW
			1210621	1230110	CCDC83	Rsb on RLW
			1236257	1253816	PICALM	Rsb on RLW
**Scaffold**	**Scaffold**	**Scaffold_Num**	**Start**	**End**	**Genes**	**Detected by**
7	NW_013185660.1	23	148419	192227	LOC106029880	hapFLK on TG
			861506	930074	LOC106029886(ARHEGF1). IQCB1. EAF2. LOC106029887	hapFLK on TG
8	NW_013185661.1	24	11450064	11452098	OTUD1	hapFLK on TG
9	NW_013185662.1		2114786	7248254	RIMS1	hapFLK on TG
15	NW_013185668.1	153	792551	797528	LOC106031663	hapFLK on TG
			805352	806966	ZFP36L1	hapFLK on TG
			808615	810911	LOC106031665	hapFLK on TG
			7699825	7713661	HEATR5A	hapFLK on TG
19	NW_013185672.1	82	2793424	2862658	NKAIN2. RNF217	hapFLK on TG
31	NW_013185684.1	368	907029	2004731	SH3BP4(negative regulator on mTOR1 which good for cell growth). Upstream. gene is LOC106034582	hapFLK on TG
33	NW_013185686.1	2	89358	897517	LOC106034864. its downstream is SLC35B3	hapFLK on TG
58	NW_013185711.1	55	1544350	1616383	LOC106037447	hapFLK on TG

			1911468	1938152	PCDH19	hapFLK on TG
			3762923	3776908	HS6ST2	hapFLK on TG
			5060563	5064141	HTR2C	hapFLK on TG
			5144084	5148021	DLG3	hapFLK on TG
			5201835	5238320	TEX11	hapFLK on TG
			5238916	5241391	LOC106037398	hapFLK on TG
85	NW_013185738.1	47	4026163	4027278	SERPINB5	hapFLK on TG
208	NW_013185861.1	52	58521	71519	TNS1	hapFLK on TG
			564853	627818	LOC106045902. LOC106045903. LOC106045915	hapFLK on TG

Table S4 Customized scaffold number by sorting scaffold length in decreasing order

Chr	Sca No.*	RefSeq.Accn	Chr	Sca No.	RefSeq.Accn	Chr	Sca No.	RefSeq.Accn	Chr	Sca No.	RefSeq.Accn
1	65	NW_013185654.1	89	135	NW_013185742.1	188	489	NW_013185841.1	290	279	NW_013185943.1
2	45	NW_013185655.1	90	8	NW_013185743.1	189	124	NW_013185842.1	291	420	NW_013185944.1
3	123	NW_013185656.1	92	128	NW_013185745.1	190	263	NW_013185843.1	292	144	NW_013185945.1
4	105	NW_013185657.1	93	72	NW_013185746.1	191	285	NW_013185844.1	293	364	NW_013185946.1
5	98	NW_013185658.1	94	306	NW_013185747.1	193	268	NW_013185846.1	295	466	NW_013185948.1
6	88	NW_013185659.1	95	211	NW_013185748.1	194	446	NW_013185847.1	296	357	NW_013185949.1
7	23	NW_013185660.1	96	15	NW_013185749.1	195	223	NW_013185848.1	298	350	NW_013185951.1
8	24	NW_013185661.1	97	157	NW_013185750.1	196	309	NW_013185849.1	299	380	NW_013185952.1
9	70	NW_013185662.1	98	222	NW_013185751.1	198	198	NW_013185851.1	300	599	NW_013185953.1
10	37	NW_013185663.1	99	154	NW_013185752.1	200	409	NW_013185853.1	301	129	NW_013185954.1
11	61	NW_013185664.1	100	18	NW_013185753.1	201	19	NW_013185854.1	303	91	NW_013185956.1
12	22	NW_013185665.1	101	245	NW_013185754.1	202	410	NW_013185855.1	305	348	NW_013185958.1
13	252	NW_013185666.1	102	21	NW_013185755.1	203	482	NW_013185856.1	307	465	NW_013185960.1
14	58	NW_013185667.1	103	188	NW_013185756.1	204	131	NW_013185857.1	309	404	NW_013185962.1
15	153	NW_013185668.1	104	73	NW_013185757.1	205	132	NW_013185858.1	310	561	NW_013185963.1
16	20	NW_013185669.1	105	4	NW_013185758.1	206	237	NW_013185859.1	311	289	NW_013185964.1
17	74	NW_013185670.1	106	145	NW_013185759.1	207	301	NW_013185860.1	312	499	NW_013185965.1
18	230	NW_013185671.1	107	33	NW_013185760.1	208	52	NW_013185861.1	313	427	NW_013185966.1
19	82	NW_013185672.1	108	371	NW_013185761.1	210	171	NW_013185863.1	314	438	NW_013185967.1
20	66	NW_013185673.1	109	281	NW_013185762.1	211	256	NW_013185864.1	315	325	NW_013185968.1
21	96	NW_013185674.1	110	194	NW_013185763.1	212	277	NW_013185865.1	316	11	NW_013185969.1
22	26	NW_013185675.1	111	84	NW_013185764.1	213	159	NW_013185866.1	317	3	NW_013185970.1
23	274	NW_013185676.1	112	137	NW_013185765.1	214	249	NW_013185867.1	318	412	NW_013185971.1
24	273	NW_013185677.1	113	426	NW_013185766.1	215	260	NW_013185868.1	319	352	NW_013185972.1
25	86	NW_013185678.1	114	39	NW_013185767.1	217	17	NW_013185870.1	320	750	NW_013185973.1
26	147	NW_013185679.1	115	162	NW_013185768.1	218	473	NW_013185871.1	321	449	NW_013185974.1
27	14	NW_013185680.1	116	56	NW_013185769.1	219	127	NW_013185872.1	322	331	NW_013185975.1
28	121	NW_013185681.1	117	287	NW_013185770.1	220	307	NW_013185873.1	323	113	NW_013185976.1

29	44	NW_013185682.1	118	292	NW_013185771.1	221	146	NW_013185874.1	324	374	NW_013185977.1
30	253	NW_013185683.1	119	204	NW_013185772.1	222	13	NW_013185875.1	325	77	NW_013185978.1
31	368	NW_013185684.1	120	42	NW_013185773.1	224	95	NW_013185877.1	326	501	NW_013185979.1
32	141	NW_013185685.1	121	108	NW_013185774.1	225	136	NW_013185878.1	328	664	NW_013185981.1
33	2	NW_013185686.1	122	240	NW_013185775.1	226	308	NW_013185879.1	329	487	NW_013185982.1
34	57	NW_013185687.1	124	25	NW_013185777.1	227	151	NW_013185880.1	333	621	NW_013185986.1
35	6	NW_013185688.1	125	329	NW_013185778.1	229	569	NW_013185882.1	334	612	NW_013185987.1
36	68	NW_013185689.1	126	49	NW_013185779.1	231	386	NW_013185884.1	336	666	NW_013185989.1
37	12	NW_013185690.1	127	326	NW_013185780.1	232	299	NW_013185885.1	337	477	NW_013185990.1
38	46	NW_013185691.1	128	109	NW_013185781.1	233	92	NW_013185886.1	338	294	NW_013185991.1
39	83	NW_013185692.1	129	313	NW_013185782.1	234	205	NW_013185887.1	339	417	NW_013185992.1
40	118	NW_013185693.1	130	48	NW_013185783.1	235	358	NW_013185888.1	340	548	NW_013185993.1
41	7	NW_013185694.1	132	213	NW_013185785.1	236	229	NW_013185889.1	341	234	NW_013185994.1
42	97	NW_013185695.1	133	369	NW_013185786.1	237	102	NW_013185890.1	342	457	NW_013185995.1
43	163	NW_013185696.1	134	168	NW_013185787.1	238	67	NW_013185891.1	343	592	NW_013185996.1
44	173	NW_013185697.1	135	323	NW_013185788.1	239	452	NW_013185892.1	344	448	NW_013185997.1
45	138	NW_013185698.1	136	181	NW_013185789.1	240	414	NW_013185893.1	347	196	NW_013186000.1
46	286	NW_013185699.1	138	354	NW_013185791.1	241	160	NW_013185894.1	348	444	NW_013186001.1
47	51	NW_013185700.1	139	28	NW_013185792.1	242	334	NW_013185895.1	349	455	NW_013186002.1
48	94	NW_013185701.1	140	356	NW_013185793.1	243	267	NW_013185896.1	350	360	NW_013186003.1
49	53	NW_013185702.1	141	140	NW_013185794.1	244	382	NW_013185897.1	351	156	NW_013186004.1
50	5	NW_013185703.1	142	89	NW_013185795.1	245	463	NW_013185898.1	354	701	NW_013186007.1
51	193	NW_013185704.1	143	41	NW_013185796.1	246	265	NW_013185899.1	355	675	NW_013186008.1
52	81	NW_013185705.1	144	180	NW_013185797.1	247	43	NW_013185900.1	357	391	NW_013186010.1
53	164	NW_013185706.1	145	276	NW_013185798.1	248	111	NW_013185901.1	360	502	NW_013186013.1
54	133	NW_013185707.1	146	304	NW_013185799.1	249	288	NW_013185902.1	361	282	NW_013186014.1
55	87	NW_013185708.1	147	381	NW_013185800.1	250	134	NW_013185903.1	362	416	NW_013186015.1
56	155	NW_013185709.1	148	215	NW_013185801.1	251	423	NW_013185904.1	363	537	NW_013186016.1
57	78	NW_013185710.1	149	303	NW_013185802.1	253	411	NW_013185906.1	364	142	NW_013186017.1
58	55	NW_013185711.1	150	388	NW_013185803.1	254	346	NW_013185907.1	365	670	NW_013186018.1

59	40	NW_013185712.1	152	165	NW_013185805.1	255	10	NW_013185908.1	366	519	NW_013186019.1
60	167	NW_013185713.1	155	359	NW_013185808.1	258	80	NW_013185911.1	368	647	NW_013186021.1
61	93	NW_013185714.1	157	218	NW_013185810.1	259	242	NW_013185912.1	369	321	NW_013186022.1
62	35	NW_013185715.1	158	115	NW_013185811.1	260	79	NW_013185913.1	375	488	NW_013186028.1
63	225	NW_013185716.1	159	90	NW_013185812.1	261	283	NW_013185914.1	377	564	NW_013186030.1
64	38	NW_013185717.1	160	269	NW_013185813.1	262	394	NW_013185915.1	379	435	NW_013186032.1
65	275	NW_013185718.1	161	125	NW_013185814.1	263	187	NW_013185916.1	382	611	NW_013186035.1
66	117	NW_013185719.1	162	209	NW_013185815.1	264	182	NW_013185917.1	384	349	NW_013186037.1
67	85	NW_013185720.1	163	224	NW_013185816.1	266	302	NW_013185919.1	386	500	NW_013186039.1
68	64	NW_013185721.1	165	126	NW_013185818.1	267	333	NW_013185920.1	388	312	NW_013186041.1
69	100	NW_013185722.1	166	34	NW_013185819.1	268	177	NW_013185921.1	394	257	NW_013186047.1
70	231	NW_013185723.1	167	54	NW_013185820.1	269	207	NW_013185922.1	395	560	NW_013186048.1
71	322	NW_013185724.1	168	112	NW_013185821.1	270	474	NW_013185923.1	396	347	NW_013186049.1
72	36	NW_013185725.1	169	174	NW_013185822.1	271	75	NW_013185924.1	399	872	NW_013186052.1
73	99	NW_013185726.1	170	387	NW_013185823.1	272	424	NW_013185925.1	400	241	NW_013186053.1
74	114	NW_013185727.1	172	293	NW_013185825.1	273	246	NW_013185926.1	401	610	NW_013186054.1
75	63	NW_013185728.1	173	27	NW_013185826.1	274	166	NW_013185927.1	402	625	NW_013186055.1
76	170	NW_013185729.1	174	258	NW_013185827.1	275	591	NW_013185928.1	403	515	NW_013186056.1
77	186	NW_013185730.1	175	367	NW_013185828.1	276	255	NW_013185929.1	405	513	NW_013186058.1
78	319	NW_013185731.1	176	361	NW_013185829.1	277	598	NW_013185930.1	406	517	NW_013186059.1
79	195	NW_013185732.1	177	60	NW_013185830.1	278	393	NW_013185931.1	410	784	NW_013186063.1
80	143	NW_013185733.1	178	219	NW_013185831.1	280	280	NW_013185933.1	411	373	NW_013186064.1
81	221	NW_013185734.1	179	110	NW_013185832.1	281	450	NW_013185934.1	415	650	NW_013186068.1
82	103	NW_013185735.1	180	339	NW_013185833.1	283	529	NW_013185936.1	424	441	NW_013186077.1
83	353	NW_013185736.1	181	59	NW_013185834.1	284	459	NW_013185937.1	429	550	NW_013186082.1
84	158	NW_013185737.1	182	101	NW_013185835.1	285	442	NW_013185938.1	430	418	NW_013186083.1
85	47	NW_013185738.1	183	236	NW_013185836.1	286	243	NW_013185939.1	432	315	NW_013186085.1
86	104	NW_013185739.1	184	254	NW_013185837.1	287	407	NW_013185940.1	434	568	NW_013186087.1
87	30	NW_013185740.1	185	201	NW_013185838.1	288	628	NW_013185941.1	449	577	NW_013186102.1
88	247	NW_013185741.1	186	107	NW_013185839.1	289	351	NW_013185942.1	457	451	NW_013186110.1

Table S5 Expression levels of APOB and TGH at four timepoints

TimePoints	Gene	RQ	TimePoints	Gene	RQ
OF-0	APOB	2,948	OF-0	TGH	0,474733
OF-0	APOB	1,828	OF-0	TGH	0,260882
OF-0	APOB	2,99	OF-0	TGH	0,523078
OF-0	APOB	1,899	OF-0	TGH	0,360069
OF-0	APOB	2,6	OF-0	TGH	0,213298
OF-0	APOB	2,065	OF-0	TGH	0,279072
OF-7	APOB	0,612	OF-7	TGH	0,153637
OF-7	APOB	2,157	OF-7	TGH	0,414028
OF-7	APOB	2,579	OF-7	TGH	0,087204
OF-7	APOB	1,753	OF-7	TGH	0,157056
OF-7	APOB	1,226	OF-7	TGH	0,244331
OF-7	APOB	2,111	OF-7	TGH	0,071192
OF-14	APOB	1,093	OF-14	TGH	0,016346
OF-14	APOB	1,448	OF-14	TGH	0,13922
OF-14	APOB	0,757	OF-14	TGH	0,04159
OF-14	APOB	0,591	OF-14	TGH	0,077713
OF-14	APOB	NA	OF-14	TGH	0,080841
OF-14	APOB	NA	OF-14	TGH	0,025251
OF-20	APOB	NA	OF-21	TGH	0,039869
OF-21	APOB	1,1654	OF-21	TGH	0,017776
OF-21	APOB	0,9734	OF-21	TGH	0,016736
OF-21	APOB	0,8415	OF-21	TGH	0,017808
OF-21	APOB	0,9969	OF-21	TGH	0,005536
OF-21	APOB	0,732	OF-21	TGH	0,051428

Notes: cell area was not provided here as there was too much data which would make it difficult to read.

Table S6 Phenotypes in each category groups with extreme high and low values

Groups* / Phenotypes¶	High-FLW	Low-FLW	High-RLW	Low-RLW	HTG	LTG	Before	After
**Sample Size**	25	24	22	21	14	10	15	305
**Prewt (g)**	4998.12 ± 536.47	4780.24 ± 384.95	4559.05 ± 385.58	4936.67 ± 406.09	4857.5 ± 526.64	4704.5 ± 509.75	-	4788.11 ± 436.6
**Pstwt (g)**	8444.71 ± 623.87	7532.19 ± 417.89	7742.71 ± 515.33	7666.83 ± 489.28	7844 ± 993.68	7541.9 ± 702.97	-	7885.76 ± 588.6
**OFG (g)**	3446.58 ± 347.31	2751.95 ± 337.1	3183.67 ± 453.29	2730.17 ± 303.36	2986.5 ± 654.91	2837.4 ± 570.18	-	3097.65 ± 434.23
**FLW (g)**	1504.88 ± 98.12	757.9 ± 93.88	1449.86 ± 135.75	751.03 ± 102.39	1178.65 ± 266.87	984.15 ± 141.78	-	1127.45 ± 206.65
**ABW (g)**	533.94 ± 66.84	467.79 ± 100.47	466.19 ± 76.61	462.39 ± 104.4	518.45 ± 89.21	417 ± 107.89	-	499.86 ± 83.73
**RLW**	0.18 ± 0.01	0.1 ± 0.01	0.19 ± 0.01	0.1 ± 0.01	0.15 ± 0.03	0.13 ± 0.02	-	0.14 ± 0.02
**AST (U/L)**	315.85 ± 192.22	172.29 ± 198.7	369.11 ± 349.37	193.48 ± 214.64	285 ± 300.5	99.81 ± 106.41	175.1 ± 116.2	321.0 ± 574.1
**ALT (U/L)**	73.71 ± 31.98	58.64 ± 44.88	80.76 ± 63.21	64.83 ± 49.1	101.81 ± 91.46	25.34 ± 21.13	86.0 ± 64.6	73.0 ± 79.0
**TBIL (µmol/L)**	10.96 ± 3.41	8.63 ± 5.66	10.54 ± 3.07	9.05 ± 5.88	9.14 ± 4.25	3.89 ± 2.25	113.5 ± 103.2	8.0 ± 4.3
**DBIL (µmol/L)**	7.62 ± 2.47	3.84 ± 4.14	7.62 ± 2.17	3.95 ± 4.27	6.4 ± 3.49	2.36 ± 1.82	14.3 ± 13.5	4.5 ± 3.3
**TC (mmol/L)**	8.64 ± 1.79	8.45 ± 3.08	8.56 ± 2.04	8.33 ± 3.17	10.2 ± 2.3	3.51 ± 1.57	5.8 ± 2.4	8.1 ± 2.6
**TG (mmol/L)**	6.89 ± 3.11	5.27 ± 2.62	7.75 ± 3.25	5.48 ± 2.85	23.55 ± 6.06	2.07 ± 0.13	3.3 ± 1.6	6.2 ± 2.6
**HDL (mmol/L)**	5.2 ± 1.66	5.7 ± 1.85	5.05 ± 1.51	5.53 ± 2	3.16 ± 1.42	2.51 ± 1.08	2.0 ± 0.3	5.4 ± 1.7
**TBA (µmol/L)**	100.36 ± 38.41	61.14 ± 27.51	99.15 ± 39.22	65.4 ± 33.69	80.32 ± 22.56	28.03 ± 14.79	21.9 ± 14.9	76.6 ± 32.1
**GLU (mmol/L)**	19.42 ± 11.53	24.62 ± 15.27	16.78 ± 9.17	25.04 ± 15.59	19.82 ± 10.5	18.78 ± 15.2	12.7± 4.2	17.5 ± 10.6
**LDL (mmol/L)**	3.45 ± 0.9	3.29 ± 1.54	3.53 ± 1.12	3.29 ± 1.56	4.36 ± 1.17	1.16 ± 0.53	1.0 ± 0.2	3.2 ± 1.3
**VLDL (mmol/L)**	0.6 ± 0.47	0.52 ± 0.41	0.69 ± 0.48	0.55 ± 0.42	1.19 ± 0.61	0.36 ± 0.19	1.1 ± 0.7	0.5 ± 0.4
**PIII (µg/L)**	32.25 ± 14.89	30.35 ± 9.26	28.36 ± 6.73	30.11 ± 9.76	169.01 ± 287.52	34.66 ± 8.24	37.8 ± 5.3	30.0 ± 8.3
**HA (ng/ml)**	396.84 ± 136.08	382.54 ± 180.91	357.47 ± 176.95	390.06 ± 187.37	593.71 ± 160.81	408.5 ± 154.12	264.5 ± 113.0	342.2 ± 161.9
**RAA**	4.19 ± 1.23	2.66 ± 0.84	4.27 ± 1.39	2.71 ± 0.91	3.38 ± 1.94	3.41 ± 1.16	2.55 ± 1.22	3.55 ± 1.67

* Group names of High-/Low-FLW. High-/Low-RLW. H/LTG. Before and After means extremely high/low fatty liver weight (FLW). extremely high/low ratio of FLW to body weight after overfeeding (RLW). extremely high/low lipid levels (TG shown). phenotypes before and after overfeeding. Prewt. Pstwt. OFG. FLW. ABW and RLW were not measured because these 15 geese were excluded due to technical reasons. ¶ Names of phenotypes were explained well in main document.

Table S7 Associations between variants from 7 genes with highest signals and all phenotypes

Chr*	Region	Gene	Prewt	Pstwt	OFG	FLW	ABW	RLW	AST	ALT	TBIL	DBIL	TC	TG	HDL	TBA	GLU	LDL	VLDL	PIII	HA	RSLT
14	7032880-7060961	ESRRG	0	0	0	1	1	16	0	0	0	0	0	6	0	0	0	0	0	7	0	0
61	4647533-4700148	GABRE	0	0	0	0	0	0	0	0	0	0	66	37	18	10	27	40	8	0	0	35
76	2904365-2989485	ARAP2	19	19	12	183	0	101	17	5	0	74	2	1	1	0	59	6	10	0	4	47
8	2475532-2570987	IL6	1	1	1	11	0	17	1	1	0	4	18	6	6	3	25	77	1	33	1	5
148	1064792-1096469	IQCJ	0	1	1	5	0	5	3	1	1	1	0	1	0	1	0	1	2	0	0	0
7	864602-895500	ARHGEF1	87	0	0	0	0	0	0	0	0	0	27	2	0	13	0	67	0	20	0	0
15	790000-80000	ZFP36L1	7	7	7	0	7	7	7	7	7	0	0	0	7	7	0	0	0	7	7	7
208	66395-66395	TNS1	0	1	1	0	0	1	0	0	1	1	0	0	0	1	0	0	1	1	0	0

* Scaffold number was defined in our study for the purpose to make some Manhattan plots more readable and details could be found in Table S6.

Table S8 Gene expression analysis from public RNA-Seq datasets

Gene	baseMean	baseMean_control_Sample_Landes_C0	baseMean_case_Sample_Landes_C20	foldChange	log2FoldChange	pval	padj	fpkm_Sample_Landes_C0_1	fpkm_Sample_Landes_C0_2	fpkm_Sample_Landes_C0_3	fpkm_Sample_Landes_C20_1	fpkm_Sample_Landes_C20_2	fpkm_Sample_Landes_C20_3
ABLIM2	193.17	253.61	132.74	0.52	-0.93	0.00	0.02	3.04	2.30	2.69	1.18	2.53	2.22
AFF2	307.95	300.28	315.61	1.05	0.07	0.85	0.98	0.99	1.52	1.38	1.52	2.11	2.13
ARAP2	41.25	38.01	44.48	1.17	0.23	0.60	0.83	0.20	0.36	0.23	0.18	0.53	0.64
BAZ1A	818.25	639.49	997.01	1.56	0.64	0.02	0.10	4.90	4.80	4.58	11.04	9.74	10.47
CCDC37	31.02	31.61	30.43	0.96	-0.05	1.00	1.00	0.51	0.36	0.24	0.44	0.30	0.64
CCDC83	1.70	0.00	3.40	Inf	Inf	0.08	0.25	0.00	0.00	0.00	0.12	0.00	0.02
CCDC89	3.20	5.04	1.35	0.27	-1.90	0.23	0.50	0.04	0.06	0.11	0.00	0.00	0.07
CLUAP1	123.38	103.54	143.22	1.38	0.47	0.17	0.41	0.46	0.90	0.72	1.25	0.97	1.79
CMPK1	594.21	562.76	625.65	1.11	0.15	0.68	0.88	9.34	10.54	10.89	16.37	16.75	15.16
DIRC2	177.05	184.07	170.02	0.92	-0.11	0.64	0.86	3.61	3.23	3.05	4.48	3.92	4.16
DLG2	83.04	67.98	98.10	1.44	0.53	0.13	0.34	0.27	0.34	0.31	0.55	0.62	0.76
EAF2	439.98	297.81	582.15	1.95	0.97	0.26	0.53	5.96	22.18	4.84	38.38	32.39	20.19
EIF5	5394.71	6414.79	4374.64	0.68	-0.55	0.03	0.12	161.66	132.39	144.80	135.88	149.43	133.91
ESRRG	200.73	229.53	171.92	0.75	-0.42	0.11	0.31	4.01	4.95	2.83	3.57	4.56	3.63
FITM2	201.47	132.63	270.32	2.04	1.03	0.01	0.06	1.46	1.42	1.10	3.95	4.56	2.76
GABRA3	0.72	1.24	0.20	0.16	-2.65	0.69	0.89	0.00	0.00	0.02	0.00	0.00	0.01
GABRE	0.00	0.00	0.00					0.00	0.00	0.00	0.00	0.00	0.00
GALNT15	64.98	36.53	93.42	2.56	1.35	0.00	0.00	0.17	0.16	0.25	0.76	0.63	1.13
HEATR5A	331.78	256.97	406.58	1.58	0.66	0.03	0.12	1.60	1.60	1.32	2.92	4.00	3.28
HSPBAP1	49.81	51.56	48.06	0.93	-0.10	0.89	1.00	1.37	0.93	0.83	1.18	1.34	1.61
HTT	552.12	712.58	391.67	0.55	-0.86	0.00	0.02	3.76	3.58	3.37	2.20	2.89	3.20
IL16	112.48	120.23	104.74	0.87	-0.20	0.76	0.94	1.11	1.25	0.95	0.81	1.24	2.36
IL1B	17.07	3.15	31.00	9.86	3.30	0.00	0.00	0.05	0.09	0.09	0.94	0.91	1.19
IL6	0.63	0.00	1.27	Inf	Inf	0.52	0.77	0.00	0.00	0.00	0.14	0.25	0.00
IQCB1	195.06	217.20	172.93	0.80	-0.33	0.24	0.50	1.82	1.92	1.39	1.83	2.06	2.06
IQCJ	2.76	0.36	5.16	14.42	3.85	0.04	0.16	0.00	0.00	0.00	0.03	0.01	0.06

LOC106030633	0.00	0.00	0.00					0.00	0.00	0.00	0.00	0.00	0.00
LOC106033540	1.32	0.36	2.28	6.37	2.67	0.40	0.68	0.00	0.05	0.00	0.13	0.11	0.09
LOC106039033(Cyp7a1)	52.64	64.53	40.75	0.63	-0.66	0.31	0.58	2.68	2.04	1.22	0.48	2.44	2.32
MAP7D3	456.21	407.79	504.63	1.24	0.31	0.23	0.49	3.67	3.14	2.83	5.12	4.96	7.11
MARK3	445.72	440.43	451.01	1.02	0.03	0.93	1.00	3.13	3.10	3.40	4.10	4.41	5.20
MDGA2	28.46	25.66	31.27	1.22	0.29	0.53	0.78	0.24	0.21	0.43	0.45	0.64	0.57
MOB2	234.35	280.87	187.83	0.67	-0.58	0.05	0.17	10.52	11.01	8.51	9.14	9.00	10.82
MOB3C	81.62	62.10	101.15	1.63	0.70	0.04	0.15	0.42	0.55	0.54	0.86	1.30	1.58
MYT1L	0.15	0.00	0.30	Inf	Inf	1.00	1.00	0.00	0.00	0.00	0.00	0.00	0.00
NOP14	376.22	356.98	395.45	1.11	0.15	0.69	0.89	4.43	4.62	4.43	7.44	6.37	6.75
NR1H4	673.95	1013.70	334.20	0.33	-1.60	0.00	0.00	17.45	22.23	17.30	6.95	7.95	12.10
OSBPL5	143.02	101.82	184.21	1.81	0.86	0.01	0.06	0.99	1.43	0.89	2.30	3.30	2.60
PANK1	2315.85	2573.21	2058.49	0.80	-0.32	0.17	0.40	63.60	65.74	43.26	70.90	73.08	48.74
PICALM	331.60	298.04	365.16	1.23	0.29	0.34	0.62	4.98	6.17	4.59	8.14	8.92	9.85
PLIN2	15847.47	9379.23	22315.70	2.38	1.25	0.00	0.00	134.95	111.90	148.78	515.49	439.89	365.81
SEL1L3	342.79	442.25	243.32	0.55	-0.86	0.02	0.11	4.21	7.33	4.59	2.78	5.02	4.93
SEMA5B	200.61	197.63	203.60	1.03	0.04	0.89	1.00	0.87	0.86	0.60	0.82	1.40	1.27
SLC15A2	1.41	2.01	0.80	0.40	-1.33	0.65	0.87	0.01	0.01	0.01	0.00	0.02	0.01
SLC7A3	252.61	146.09	359.14	2.46	1.30	0.01	0.05	2.91	4.69	2.28	14.09	8.38	13.38
SOD3	198.6	82.05	315.15	3.84	1.94	0.01	0.04	2.17	2.84	4.68	24.37	8.11	24.52
SORCS3	119.72	109.85	129.59	1.18	0.24	0.58	0.82	0.55	0.96	0.94	1.47	1.12	1.29
SYTL2	221.18	339.51	102.86	0.30	-1.72	0.00	0.00	1.46	1.11	1.61	0.35	0.64	0.84
TMEM126A	172.27	185.42	159.12	0.86	-0.22	0.39	0.67	14.57	14.69	13.42	20.09	11.15	16.96
TNIP2	108.57	123.36	93.78	0.76	-0.40	0.24	0.50	0.86	1.27	0.65	0.71	1.05	1.20
TNS1	633.02	623.36	642.69	1.03	0.04	0.75	0.93	3.06	3.86	5.07	4.63	5.42	8.38
VEGFA	1585.42	2188.03	982.80	0.45	-1.15	0.00	0.00	32.61	30.65	38.83	19.37	19.11	28.51
VRK1	39.83	38.87	40.79	1.05	0.07	0.89	1.00	0.75	1.20	0.58	0.56	1.54	1.38
ZFP36L1	3654.80	3564.88	3744.72	1.05	0.07	0.78	0.95	136.64	129.36	217.05	242.94	201.04	283.76

Other hundreds of genes were not shown here.

Table S9 Top 10 GO gene terms belong to Biological Process (BC) found in RNA-Seq data analysis

id	Term¶	pval	Score#	Gene	Breeds
GO:0006531	aspartate metabolic process	0	33	DDO; GOT1; GOT2	ZD*
GO:0019915	lipid storage	1.16e-07	13.2	HEXB; FITM2; ANGPTL3; DGAT2; PLIN2; LOC106038244	ZD
GO:0015909	long-chain fatty acid transport	1.30e-06	16.5	SLC27A1; PPARG; PLIN2; FABP3	ZD
GO:0031643	positive regulation of myelination	1.19e-05	16.5	NCMAP; TENM4; LOC106038521	ZD
GO:0043537	negative regulation of blood vessel endothelial cell migration	1.19e-05	16.5	HRG; THBS1; VASH1	ZD
GO:0006108	malate metabolic process	1.19e-05	16.5	MDH1; MDH2; ME1	ZD
GO:0003181	atrioventricular valve morphogenesis	1.19e-05	16.5	HEYL; CYR61; ZFPM1	ZD
GO:0010891	negative regulation of sequestering of triglyceride	1.19e-05	16.5	PPARG; ABHD5; LOC106038244	ZD
GO:0090131	mesenchyme migration	1.19e-05	16.5	ACTA1; ACTA2; TGFB3	ZD
GO:0006631	fatty acid metabolic process	1.46e-05	6.08	FA2H; ACSF3; GPAM; ANGPTL3; ABHD5; AACS; FABP3	ZD
GO:0015760	glucose-6-phosphate transport	0	15.25	LOC106029669; G6PC; SLC37A4	LD*
GO:0006642	triglyceride mobilization	0	15.25	APOB; SIRT1; LPIN1	LD
GO:0045922	negative regulation of fatty acid metabolic process	0	15.25	LOC106034450; LOC106034451	LD
GO:0045939	negative regulation of steroid metabolic process	0	15.25	LOC106034450; LOC106034451	LD
GO:1904224	negative regulation of glucuronosyltransferase activity	0	15.25	LOC106034450; LOC106034451	LD
GO:0009308	amine metabolic process	0	15.25	LOC106048001; LOC106048002	LD
GO:0006958	complement activation. classical pathway	4.78e-12	9.01	MASP2; C3; C5; C6; C9; C1R; C1S; C8A; C8B; C8G; CFI	LD
GO:0006957	complement activation. alternative pathway	1.05e-09	11.09	C3; C5; C9; LOC106041557; C8A; C8B; C8G; CFH	LD
GO:0044281	small molecule metabolic process	2.47e-09	2.03	HPD; PLD4; PHKA2; CA5A; INPP5B; NT5C2; GART; SULT2B1; APOB; OAT; ACSL1; PPIP5K2; NCOA1; NCOA2; GCLC; ACSS2; MINPP1; DDC; HLCS; MOCOS; PPAT; PCK1; IP6K2; ATIC	LD
GO:0006956	complement activation	3.96e-09	10.17	LOC106043254; MASP2; C3; LOC106044835; C1S; C8A; C8B; CFH	LD

* ZD and LD refers to Zhedong White Goose and Landes Goose breeds. ¶ Terms highlighted by green color are associated with lipid mobilization and accumulation in lipid droplets. # Score is short for enrichment score for GO terms.

Table S10 Effects of sex, house, and workers on phenotypes*

Phenotypes \ Factors	Sex (p values)	Worker (p values)	Farm house (p values)
RLW	1.17% (0.011)	3.82% (1.06e-4)	3.37% (3.98e-5)
FLW	0.17% (0.65)	1.17% (0.038)	0.61% (0.05)
Pstwt	6.11% (3.81e-8)	2.56% (1.8e-3)	2.63% (2.6e-4)
Prewt	20.44% (<2.2-16)	1.52% (0.018)	1.64% (3.3e-3)

*A linear model was used to estimate the effects from sex, worker and farm house on different phenotypes.
